# Bactericidal fully human single‐chain fragment variable antibodies protect mice against methicillin‐resistant *Staphylococcus* *aureus* bacteraemia

**DOI:** 10.1002/cti2.1302

**Published:** 2021-06-29

**Authors:** Behnoush Soltanmohammadi, Somayeh Piri‐Gavgani, Eilnaz Basardeh, Mostafa Ghanei, Masoumeh Azizi, Zabihollah Khaksar, Zahra Sharifzadeh, Farzad Badmasti, Mahdieh Soezi, Abolfazl Fateh, Parisa Azimi, Seyed Davar Siadat, Fahimeh Shooraj, Saeid Bouzari, Mir Davood Omrani, Fatemeh Rahimi‐Jamnani

**Affiliations:** ^1^ Department of Mycobacteriology and Pulmonary Research Pasteur Institute of Iran Tehran Iran; ^2^ Microbiology Research Center Pasteur Institute of Iran Tehran Iran; ^3^ Chemical Injuries Research Center Systems Biology and Poisoning Institute Baqiyatallah University of Medical Sciences Tehran Iran; ^4^ Molecular Medicine Department, Biotechnology Research Center Pasteur Institute of Iran Tehran Iran; ^5^ Department of Basic Sciences School of Veterinary Medicine Shiraz University Shiraz Iran; ^6^ Department of Immunology Pasteur Institute of Iran Tehran Iran; ^7^ Department of Bacteriology Pasteur Institute of Iran Tehran Iran; ^8^ Molecular Biology Department Pasteur Institute of Iran Tehran Iran; ^9^ Department of Medical Genetics School of Medicine Shahid Beheshti University of Medical Sciences Tehran Iran

**Keywords:** bacteraemia, bactericidal antibodies, fully human antibody, methicillin‐resistant *Staphylococcusaureus*, single‐chain fragment variable

## Abstract

**Objectives:**

The increasing prevalence of antibiotic‐resistant *Staphylococcus aureus*, besides the inadequate numbers of effective antibiotics, emphasises the need to find new therapeutic agents against this lethal pathogen.

**Methods:**

In this study, to obtain antibody fragments against *S. aureus*, a human single‐chain fragment variable (scFv) library was enriched against living methicillin‐resistant *S. aureus* (MRSA) cells, grown in three different conditions, that is human peripheral blood mononuclear cells with plasma, whole blood and biofilm. The antibacterial activity of scFvs was evaluated by the growth inhibition assay *in vitro*. Furthermore, the therapeutic efficacy of anti‐*S*. *aureus* scFvs was appraised in a mouse model of bacteraemia.

**Results:**

Three scFv antibodies, that is MEH63, MEH158 and MEH183, with unique sequences, were found, which exhibited significant binding to *S*. *aureus* and reduced the viability of *S*. *aureus* in *in vitro* inhibition assays. Based on the results, MEH63, MEH158 and MEH183, in addition to their combination, could prolong the survival rate, reduce the bacterial burden in the blood and prevent inflammation and tissue destruction in the kidneys and spleen of mice with MRSA bacteraemia compared with the vehicle group (treated with normal saline).

**Conclusion:**

The combination therapy with anti‐*S*. *aureus* scFvs and conventional antibiotics might shed light on the treatment of patients with *S. aureus* infections.

## Graphical abstract

In this study, we found that MEH63, MEH158 and MEH183 scFvs, with unique sequences, exhibited significant binding to *Staphylococcus aureus* and reduced the viability of *S. aureus*.

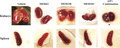

## Introduction

*Staphylococcus aureus* is one of the most well‐adapted pathogens, found as a commensal microorganism in more than one‐third of the world’s population.^1^ This pathogen, by disseminating into the bloodstream, causes a group of complicated infections, such as endocarditis, osteomyelitis, pneumonia and bacteraemia, particularly in high‐risk individuals (e.g. immunocompromised patients and infants).^2^, ^3^, ^4^, ^5^ Among *S*. *aureus*‐associated infections, bacteraemia, with an annual occurrence of 10–30 per 100 000 people and a mortality rate of approximately 40% in developed countries, is a serious clinical concern.^6^ The emergence of methicillin‐resistant *S*. *aureus* (MRSA) strains has caused challenges in treating patients with bacteraemia, as only a few antibiotics, such as vancomycin and daptomycin, remain effective,^7^ even some MRSA strains have shown resistance to these two antibiotics.^4^, ^8^ Considering the existence of MRSA persisters, the formation of strong biofilms by MRSA strains (resulting in chronic and recurrent/relapsing infections resistant to routine treatment), the costly production of novel antibiotics and the emergence of resistant *S. aureus* strains, some pharmaceutical companies have attempted to find new antibiotics.^9^, ^10^

*Staphylococcus aureus* has a multifaceted cell wall, consisting of cell wall‐anchored proteins, wall teichoic acids, lipoteichoic acids and polysaccharides, which helps the pathogen to interact with the host, evade the immune response and develop infections.^10^, ^11^, ^12^, ^13^, ^14^ It seems that targeting multiple surface virulence factors of *S*. *aureus* by therapeutics such as monoclonal antibodies (mAbs) with specific binding abilities and effector functions can be a complex strategy, not only inhibiting the growth and pathogenicity of the bacterium, but also preventing the emergence of resistant strains.^3^, ^15^, ^16^ So far, a group of mAbs, such as Altastaph, Veronate, Tefibazumab, Pagibaximab and Aurograb, has been introduced. Although these mAbs were found to be successful in animal models of infection, they lacked efficacy in clinical trials.^3^, ^16^, ^17^, ^18^, ^19^, ^20^, ^21^, ^22^ Nevertheless, several studies and projects are underway to develop functional mAbs,^23^, ^24^, ^25^ among which MEDI6389 targeting multiple components of *S. aureus* (alpha toxin, clumping factor A [ClfA], leucocidin SF, leucotoxin ED and gamma‐haemolysin AB and CB),^24^ DSTA4637S targeting β‐N‐acetylglucosamine conjugated with rifamycin,^6^ and 514G3 against staphylococcal protein A (SpA) (Fc region of immunoglobulin G3 [IgG3] not recognised by SpA)^26^ have shown promising results in preclinical studies. It is worth mentioning that these antibodies have drawbacks such as high‐cost production, low tissue penetration and Fc‐related side effects, affecting their development and application.^27^ In recent decades, particular attention has been paid to antibody fragments, either as single molecules or in intricate structures (e.g. bispecific fragments), against targets associated with cancers, autoimmune disorders and infectious diseases.^28^, ^29^, ^30^ Among antibody fragments, the single‐chain fragment variable (scFv), consisting of heavy‐ and light‐chain variable domains of an antibody (VH and VL respectively) joined by a peptide linker, has become increasingly popular for research laboratories and clinical applications because of its small size, binding ability and low immunogenicity.^28^, ^29^, ^30^, ^31^ Moreover, the scFv fragment can be expressed in various hosts, making it possible to produce large quantities easily and cost‐effectively.^28^, ^29^, ^30^ A group of scFvs has been generated against pathogen targets,^32^ some of which showed direct bactericidal activities.^33^, ^34^, ^35^, ^36^ These antibacterial scFvs seem to exert their bactericidal effects by disrupting the bacterium’s biological activity, compromising the cell wall integrity or functioning as abzymes.^35^, ^37^, ^38^, ^39^ In this regard, Wang *et al*. isolated eight high‐affinity anti‐*S*. *aureus* scFvs from a phage‐display library, which was constructed from the peripheral blood lymphocytes of cows with mastitis caused by *S. aureus*.^36^ They found that the eight anti‐*S*. *aureus* scFvs not only inhibited the growth of *S. aureus in vitro*, but also exerted a protective effect in a murine model of *S. aureus* mastitis.^36^

In the present study, to isolate scFv‐specific *S. aureus*, a fully human scFv phage library was enriched against living MRSA strains, which were cultured in different conditions, resembling their growth conditions in the human body and biofilm. Three scFv antibodies, which could recognise and inhibit the growth of *S. aureus in vitro*, were identified. These anti‐*S*. *aureus* scFvs (alone and in combination) demonstrated therapeutic efficacy in a mouse model of bacteraemia.

## Results

### Antibiotic susceptibility

The antibiotic susceptibility of *S. aureus* S.a.48, S.a.61, S.a.124 and ATCC 6538 was tested using the minimum inhibitory concentration (MIC) test strip assay. Based on the results, only the ATCC 6538 strain showed susceptibility to oxacillin (MIC = 0.2 µg mL^−1^), and no inhibition zone was detected for *S*. *aureus* S.a.48, S.a.61 and S.a.124 (MIC > 256 µg mL^−1^; extremely resistant to oxacillin) (Table 1). According to the broth microdilution assay, *S. aureus* S.a.48, S.a.61, S.a.124 and ATCC 6538 and *Staphylococcus epidermidis* ATCC 12228 showed susceptibility to vancomycin (MICs ranged from 1 to 2 µg mL^−1^). *Streptococcus pyogenes* ATCC 10403 was susceptible to ampicillin (MIC = 0.5 µg mL^−1^) (Table 1).

**Table 1:** Minimum inhibitory concentrations (MICs) of oxacillin, vancomycin and ampicillin against the studied bacteria

Strain	Antibiotic	MIC (µg mL^‐1^)
*S. aureus* S.a.48	Oxacillin	> 256
*S. aureus* S.a.61		> 256
*S. aureus* S.a.124		> 256
*S. aureus* ATCC 6538		0.2
*S. aureus* S.a.48	Vancomycin	2
*S. aureus* S.a.61		2
*S. aureus* S.a.124		2
*S. aureus* ATCC 6538		1
*S. epidermidis* ATCC 12228		1
*S. pyogenes* ATCC 10403	Ampicillin	0.5

### Biofilm formation

The biofilm formation ability of the MRSA strains (*S. aureus* S.a.48, S.a.61 and S.a.124) and one methicillin‐susceptible *S. aureus* (MSSA) strain (*S. aureus* ATCC 6538) was assessed using the crystal violet staining assay. The average optical density at 595 nm (OD_595_) for *S*. *aureus* S.a.48, S.a.61, S.a.124 and ATCC 6538 was 1.1, 1.3, 1.2 and 1.4 after 24 h and 2.02, 2.22, 2.15 and 2.4 after 72 h respectively; therefore, all four strains were strong biofilm producers (Supplementary figure 1). The MSSA strain and *S*. *aureus* S.a.61 (isolated from an intravascular catheter) showed the highest biofilm formation ability, indicating the effects of antibiotic susceptibility and isolation origin.

### Isolation of *S. aureus*‐specific phages

The biopanning procedure was carried out in three separate lines, which differed in terms of growth conditions of bacteria. Phages displaying scFvs were incubated with living bacteria, grown in human peripheral blood mononuclear cells (PBMCs) with plasma, in whole blood or as a biofilm. The polyclonal assay was carried out to determine which round of biopanning contained the pool of phages with strong binding abilities to *S*. *aureus*. The results showed that the output phages of the third round of PBMC–plasma, blood and biofilm biopanning had the greatest binding to *S. aureus* (data not shown). Therefore, 140, 200 and 200 colonies containing the output phages of the third round of PBMC–plasma, blood and biofilm biopanning, respectively, were selected randomly and assessed with regard to their binding ability to *S. aureus*. For further evaluations, we selected 11 phage clones (MEH63, MEH79 and MEH94 from PBMC–plasma biopanning; MEH121, MEH131, MEH169 and MEH178 from blood biopanning; and MEH158, MEH183, MEH188 and MEH199 from biofilm biopanning), which showed the strongest binding to *S. aureus* relative to the controls (Supplementary figure 2).

### Production of soluble scFv antibodies

**Figure 1:**
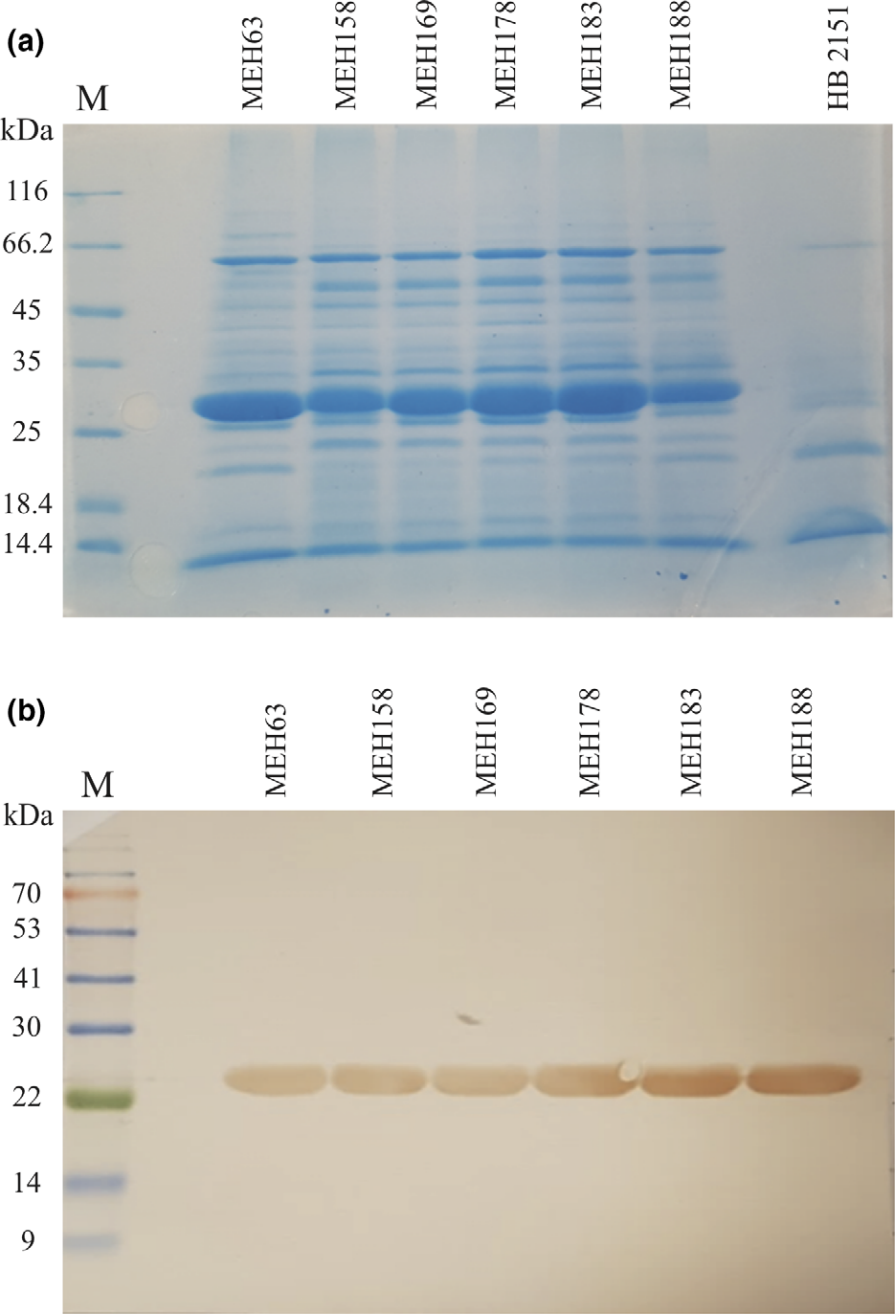
scFv antibodies were expressed in *E*. *coli* HB2151. **(a)** The successful expression of scFv antibody (molecular weight, 27 kDa) in *E. coli* HB2151 was examined by SDS‐PAGE (12%). Lane M, protein marker; lane HB2151, the periplasmic extract of normal *E. coli* HB2151 induced by 0.1 mM IPTG. **(b)** Western blot analysis. A single band of approximately 27 kDa, corresponding to scFv, was found by probing with mouse anti‐human scFv antibody, followed by goat anti‐mouse IgG‐horseradish peroxidase (HRP) antibody, and visualised by DAB. Lane M, protein marker. DAB, diaminobenzidine; IPTG, isopropyl β‐d‐1‐thiogalactopyranoside.

The presence of scFvs in the periplasm of *Escherichia coli* HB2151, which was individually infected with phages derived from 11 phage clones, was assessed by sodium dodecyl sulphate–polyacrylamide gel electrophoresis (SDS‐PAGE). Based on the results, six clones, including MEH63, MEH158, MEH169, MEH178, MEH183 and MEH188, with an expression yield of approximately 1 mg mL^−1^ were selected (Figure 1a). In the Western blot analysis, a band of about 27 kDa was detected, which indicated the successful expression of soluble scFv antibodies (Figure 1b).

### Identification of *S. aureus*‐specific scFvs

**Figure 2:**
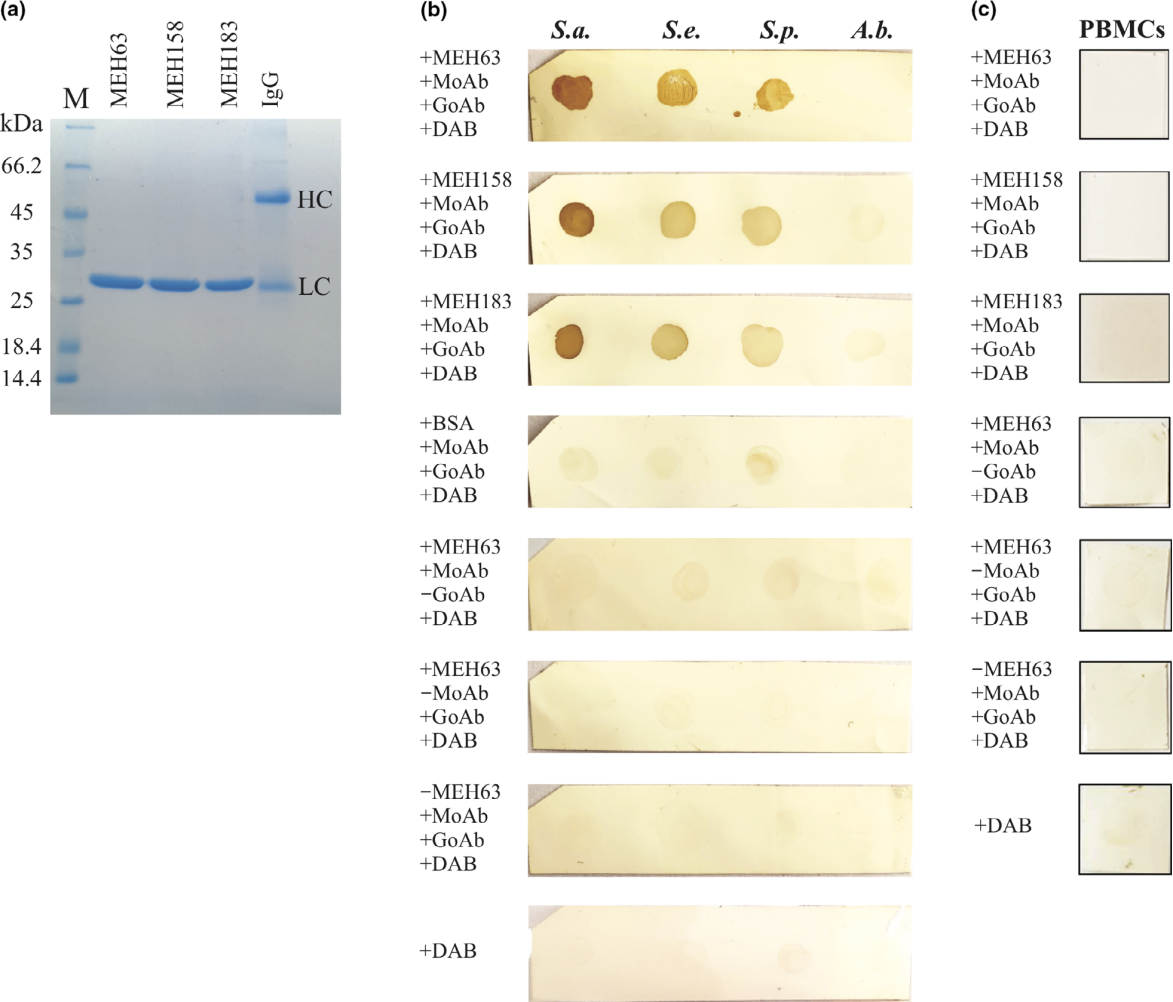
MEH63, MEH158 and MEH183 showed significant binding to *S*. *aureus*. The purity and binding ability of MEH63, MEH158 and MEH183 were analysed by reducing SDS‐PAGE (12%) and dot‐blot assay respectively. **(a)** SDS‐PAGE. A single band was observed at ~ 27 kDa. Lane M, protein marker. **(b)** The binding ability of MEH63, MEH158 and MEH183 to *S. aureus* S.a.124 (*S.a*.), *S. epidermidis* ATCC 12228 (*S.e*.), *S. pyogenes* ATCC 10403 (*S.p*.) and *A. baumannii* A.b.56 (*A.b*.) was appraised by the dot‐blot assay. The controls included the spotted bacteria incubated with BSA (5 mg mL^‐1^), followed by mouse anti‐human scFv antibody (MoAb) and then goat anti‐mouse IgG‐horseradish peroxidase (HRP) antibody (GoAb); MEH63 and then mouse anti‐human scFv antibody; MEH63 and then goat anti‐mouse IgG‐HRP; mouse anti‐human scFv antibody and then goat anti‐mouse IgG‐HRP; and DAB. **(c)** The cross‐reactivity of scFvs with PBMCs was examined using the dot‐blot assay. The controls included the cells incubated with MEH63 and then mouse anti‐human scFv antibody; MEH63 and then goat anti‐mouse IgG‐HRP; mouse anti‐human scFv antibody and then goat anti‐mouse IgG‐HRP; and DAB. BSA, bovine serum albumin; DAB, diaminobenzidine; IgG, immunoglobulin G; LC, light chain; HC, heavy chain; PBMCs, peripheral blood mononuclear cells.

The sequence analysis of six scFvs revealed that MEH63, MEH158 and MEH183 shared common sequences with MEH188, MEH178 and MEH169 respectively (Supplementary figure 3). The nucleotide sequences of the three selected scFvs, including MEH63, MEH158 and MEH183, were further evaluated in the IMGT/V‐QUEST database; the results showed that the VH and VL domains of the selected scFvs were rearranged from the human IGHV1‐46*01 F germline genes (with a complementarity determining region 3 [CDR3] length of 14 amino acids) and human IGKV1‐39*01 F germline genes (with a CDR3 length of 9 amino acids) respectively. To assess the binding ability of MEH63, MEH158 and MEH183 to *S*. *aureus*, the scFvs were purified by an immobilised metal affinity chromatography (IMAC) and then dialysed. The purity of scFvs was examined by SDS‐PAGE, demonstrating a single band at approximately 27 kDa (Figure 2a). In a dot‐blot assay, all three scFvs (MEH63, MEH158 and MEH183) exhibited strong binding to *S*. *aureus* S.a.124 and moderate binding to *S. epidermidis* ATCC 12228 and *S. pyogenes* ATCC 10403 (Figure 2b). None of the scFvs showed any binding activity with *Acinetobacter baumannii* A.b.56 (Figure 2b). As presented in Figure 2c, MEH63, MEH158 and MEH183 exhibited no off‐target binding to PBMCs.

### Significant inhibitory activities of MEH63, MEH158 and MEH183 against *S. aureus*

First, the antibacterial activity of vancomycin (0.0625–32 µg mL^−1^) against *S*. *aureus* S.a.48, S.a.61, S.a.124 and ATCC 6538 was assessed using the microtitre plate assay, which demonstrated growth inhibitory activities at concentrations above 1 µg mL^−1^ (Supplementary figure 4). Based on the microtitre plate assay, MEH63, MEH158 and MEH183 (200 µg mL^−1^) could significantly affect the growth curves of *S*. *aureus* S.a.48, S.a.61, S.a.124 and ATCC 6538 compared with the growth curves of untreated bacteria and bacteria treated with denatured MEH158 scFv (Supplementary figure 5). Based on the results, vancomycin exerted the most significant inhibitory effects on the growth of *S*. *aureus* strains, followed by the scFvs.

**Figure 3:**
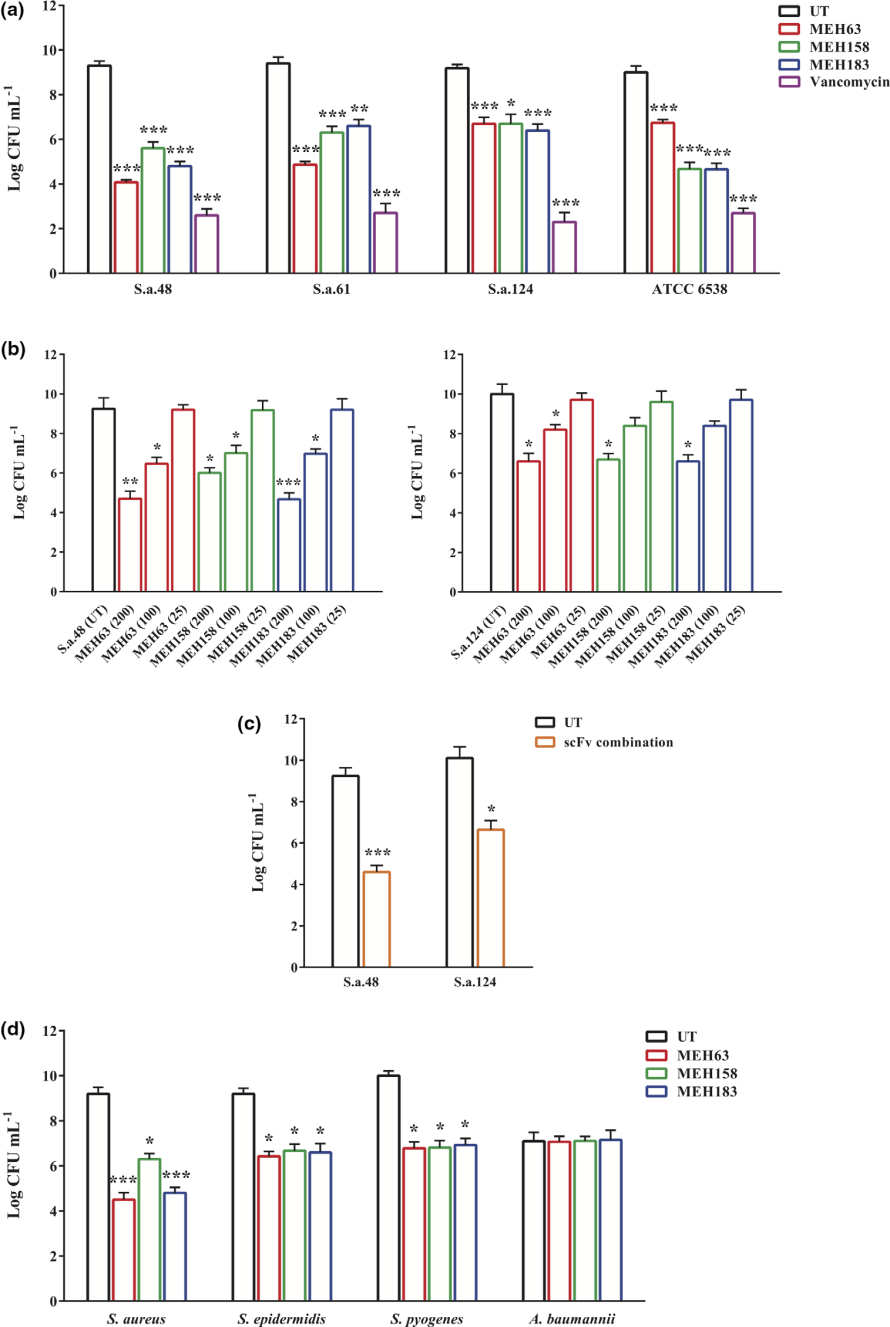
MEH63, MEH158 and MEH183 exhibited antibacterial activities. The antimicrobial activity of MEH63, MEH158 and MEH183 was evaluated by the agar plate assay. **(a)** The inhibitory activity of MEH63, MEH158 and MEH183 against S. *aureus* S.a.48, S.a.61, S.a.124 and ATCC 6538 (after 4 h of incubation). Vancomycin was used as the positive control. **(b)** Different concentrations of MEH63, MEH158 and MEH183, and **(c)** the scFv combination could significantly reduce the colony‐forming unit count of *S*. *aureus* S.a.48 and S.a.124. **(d)** MEH63, MEH158 and MEH183 (200 µg mL^‐1^) exhibited antibacterial activity against S. *aureus* S.a.48, *S*. *epidermidis* ATCC 12228 and *S*. *pyogenes* ATCC 10403, but not against *A*. *baumannii* A.b.56. Data are representative of three independent experiments, and error bars correspond to the mean ± SEM. * *P*‐value < 0.05, ** *P*‐value = 0.01 and *** *P*‐value < 0.01.

In the agar plate assay, vancomycin (at concentrations ≥1 µg mL^−1^) (data not shown) and all three scFvs (200 µg mL^−1^) showed bactericidal activities and decreased the viability of *S*. *aureus* S.a.48, S.a.61, S.a.124 and ATCC 6538 compared with untreated bacteria (Figure 3a). Regarding the untreated bacteria, although the MEH63 scFv exhibited the highest reduction in the viability of *S*. *aureus* S.a.48 (44% viability) and *S. aureus* S.a.61 (52% viability), the antibacterial activity against *S*. *aureus* S.a.124 and ATCC 6538 was low (approximately 72% viability). However, MEH158 and MEH183 showed similar bactericidal activities and decreased the viability of *S. aureus* S.a.48, S.a.61, S.a.124 and ATCC 6538 by approximately 40%, 30%, 30% and 50% respectively.

To investigate whether MEH63, MEH158 and MEH183 function in a concentration‐dependent manner, *S. aureus* S.a.48 and S.a.124 were individually treated with 25, 100 and 200 µg mL^−1^ of scFv (Figure 3b). Based on the results, the bactericidal efficacy of MEH63, MEH158 and MEH183 against *S. aureus* S.a.48 and S.a.124 was directly associated with the concentration of scFvs.

The antibacterial effects of the combination of three scFvs on *S. aureus* S.a.48 and S.a.124 were also assessed, and the results demonstrated its significant impact on the viability of MRSA bacteria (50% and 65% viability respectively) (Figure 3c).

Moreover, the antibacterial activities of MEH63, MEH158 and MEH183 (200 µg mL^−1^) were examined against *S*. *epidermidis* ATCC 12228, *S*. *pyogenes* ATCC 10403 and *A*. *baumannii* A.b.56. The results indicated about a 30% reduction in the viability of two Gram‐positive bacteria and no significant effect on the growth of *A. baumannii* (Figure 3d).

### Targeting *S. aureus* by MEH63, MEH158 and MEH183

**Figure 4:**
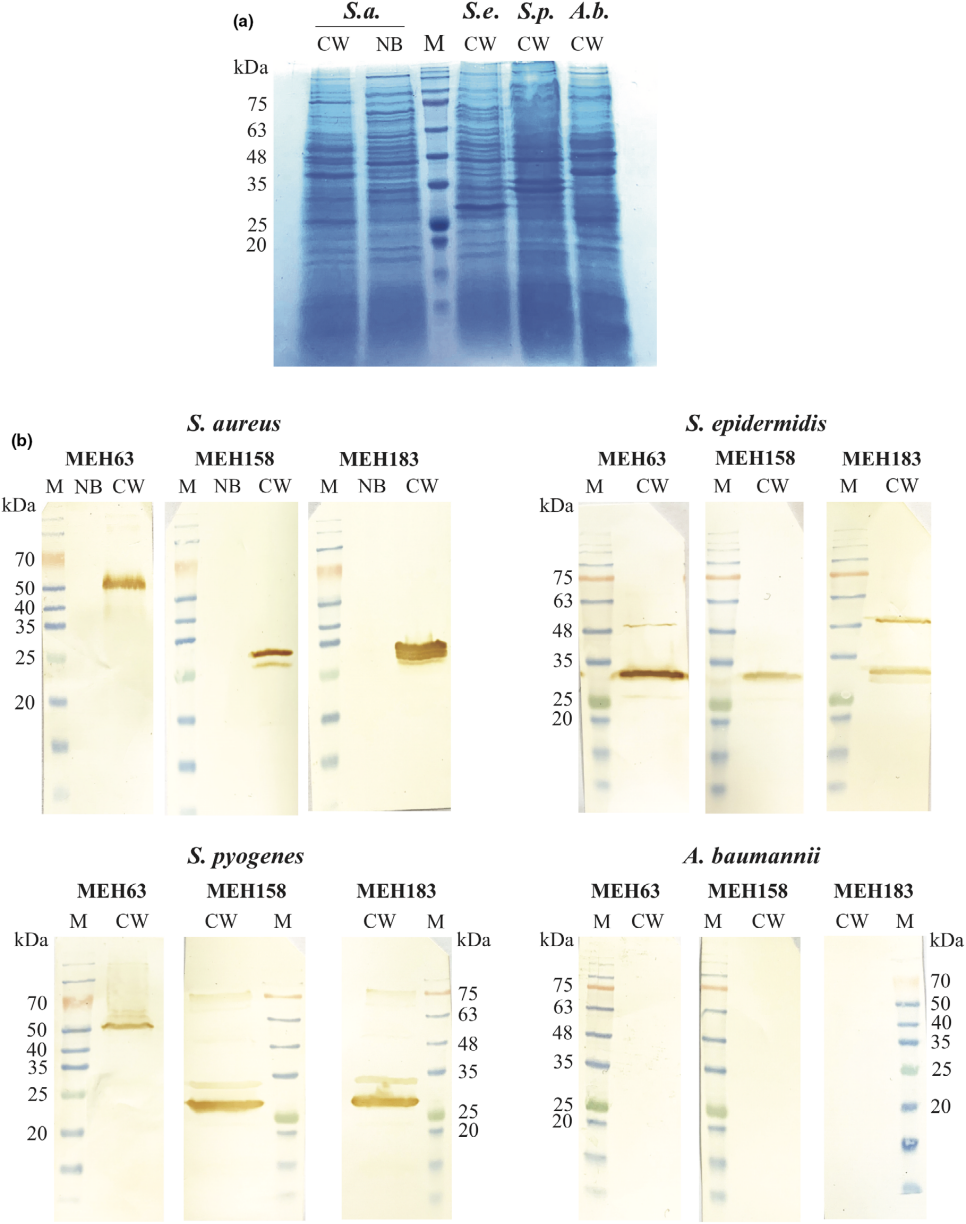
MEH63, MEH158 and MEH183 reacted with some cell wall proteins of *S. aureus, S. epidermidis* and *S. pyogenes*. The crude cell wall extracts (CW) of *S. aureus* S.a.124 (*S.a*.)*, S. epidermidis* ATCC 12228 (*S.e*.), *S. pyogenes* ATCC 10403 (*S.p*) and *A. baumannii* A.b.56 (*A.b*.), as well as non‐covalent bond cell wall proteins (NB) of *S. aureus* S.a.124, were assessed by SDS‐PAGE and Western blot analysis. **(a)** SDS‐PAGE (12%). Lane M, protein marker. **(b)** Western blot analysis. The crude cell wall extracts and non‐covalent bond cell wall proteins, separated by SDS‐PAGE, were blotted onto the PVDF membranes. The membranes were incubated with MEH63, MEH158 or MEH183. After incubation with mouse anti‐human scFv antibody and then goat anti‐mouse IgG‐horseradish peroxidase (HRP) antibody, the bands were visualised with DAB and H_2_O_2_. Lane M, protein marker. DAB, diaminobenzidine.

Following the enrichment of a peptide‐phage library against MEH63, MEH158 and MEH183, the phage DNA of 12 phage clones was purified and sequenced. Among 12 phage clones, all four MEH63‐related clones (‐CRSPDNYPC‐; 100%), three of four MEH158‐related clones (‐CMARYMSAC‐; 75%) and two of four MEH183‐related clones (‐CMARYMSAC‐; 50%), encoding peptides with seven residues, flanked with two cysteines (C‐X7‐C),^40^ were selected for more evaluations. The selected sequences were checked in the MimoDB database, and the results showed that all peptides were target‐true binders. The MEH63‐, MEH158‐ and MEH183‐specific peptide sequences were blasted against the NCBI protein database for *S. aureus*, *S. epidermidis* and *S. pyogenes,* and the predicted proteins were assessed in the UniProt database. The crude cell wall extracts of *S. aureus* S.a.124, *S. epidermidis* ATCC 12228, *S. pyogenes* ATCC 10403 and *A. baumannii* A.b.56, as well as non‐covalent bond cell wall proteins of *S. aureus* S.a.124, were assessed by SDS‐PAGE and Western blot analysis (Figure 4a and b). Based on the results of Western blot analysis on the cell wall extract of *S. aureus,* MEH63 identified a band between 50 and 55 kDa, corresponding to the TrkH family potassium uptake protein (~50 kDa), predicted from the MEH63‐specific peptide (‐CRSPDNYPC‐) (Figure 4b). Moreover, MEH158 and MEH183 detected the peptidoglycan editing factor (PgeF; ~30 kDa) and lipoprotein‐like 8 (lpl8; ~31 kDa), predicted from the MEH158/MEH183‐specific peptide (‐CMARYMSAC‐) (Figure 4b).

Based on the Western blot analysis of the cell wall extracts of *S. epidermidis* and *S. pyogenes,* MEH63 detected the *S. epidermidis* protein with a molecular weight of about 35 kDa and the *S. pyogenes* protein with a molecular weight of about 55 kDa, which were close to the molecular weights of candidate proteins, including ATP‐binding cassette (ABC) transporter substrate‐binding proteins (~37 kDa and ~55 kDa respectively), predicated from the MEH63‐specific peptide (Figure 4b). MEH158 and MEH183 detected a band between 25 and 35 kDa, corresponding to the PgeF of *S. epidermidis* (~30 kDa) (Figure 4b). Besides, MEH183 detected a band about 48 kDa, close to the molecular weight of the ABC transporter permease (~48 kDa) (Figure 4b). Also, MEH158 and MEH183 identified a sharp band between 25 and 30 kDa, corresponding to the D‐alanyl‐D‐alanine carboxypeptidase of *S. pyogenes* (~27 kDa). Although MEH158 and MEH183 detected a faint band between 30 and 35 kDa, no *S. pyogenes* proteins were found at this molecular weight among candidate proteins, predicated from the MEH158/MEH183‐specific peptide (Figure 4b).

### Amino acid sequence analysis

After analysing the CDRs of MEH63, MEH158 and MEH183 in the Antimicrobial Peptide Database (APD), no CDRs were found as AMPs already registered in the database. Furthermore, the negative grand average of hydropathicity (GRAVY) scores demonstrated the hydrophilic entity of CDRs. However, as MEH63, MEH158 and MEH183 were significantly positively charged proteins (isoelectric point [pI] of 9.14, 9.37 and 9.26 respectively), it was hypothesised they might exert their bactericidal effects as cationic AMPs.

### Inhibition of the bactericidal activity of MEH63, MEH158 and MEH183 by Mg^2+^

The treatment of *S*. *aureus* S.a.48, S.a.61, S.a.124 and ATCC 6538, *S. epidermidis* ATCC 12228 and *S. pyogenes* ATCC 10403 with MEH63, MEH158 and MEH183 in the presence of high concentrations of Mg^2+^ resulted in no growth inhibition at 30 min and 4 h of incubation compared with the controls (Supplementary figures 6–17). Consequently, it can be proposed that bulky cationic scFvs compromised the cell wall integrity by competing with Mg^2+^ in binding to teichoic acids^41^, ^42^, ^43^ and also by binding to their targets, resulting in a significant growth inhibitory activity.^44^

### Additive effects between anti‐*S. aureus* scFvs and vancomycin

The effects of the combination of anti‐*S*. *aureus* scFvs and vancomycin on the growth of MRSA (*S*. *aureus* S.a.48 and S.a.124) were determined using the checkerboard assay. Additivity was observed in all four combinations (MEH63, MEH158, MEH183 and a cocktail of three scFvs in combination with vancomycin) against *S*. *aureus* S.a.48 and S.a.124 (Table 2). Overall, the present results revealed that the combination of vancomycin and anti‐*S*. *aureus* scFvs could lead to a more significant bactericidal activity against *S*. *aureus* compared with their sole use.

**Table 2:** Fractional inhibitory concentration index (FICI) values of MEH63, MEH158, MEH183 and a cocktail of three scFvs in combination with vancomycin

Strain	Combination	FICI	Effect
*S. aureus* S.a.48	MEH63/vancomycin	0.9	Additive
	MEH158/vancomycin	0.7	
	MEH183/vancomycin	0.9	
	a cocktail of three scFvs/vancomycin	0.8	
*S. aureus* S.a.124	MEH63/vancomycin	0.9	Additive
	MEH158/vancomycin	0.7	
	MEH183/vancomycin	0.9	
	a cocktail of three scFvs/vancomycin	0.8	

### Haemolytic and cytotoxic activities of anti‐*S. aureus* scFvs

**Figure 5:**
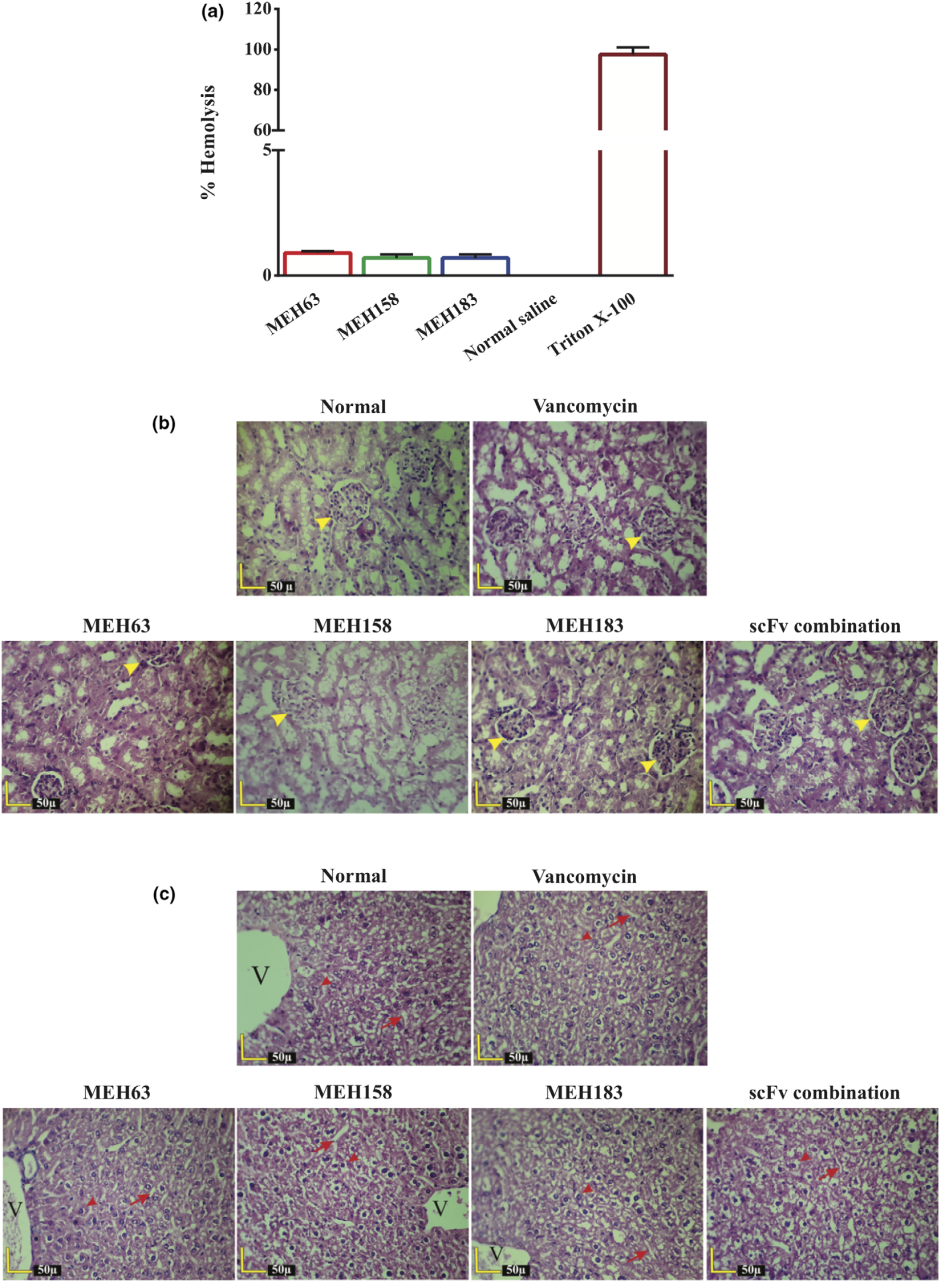
MEH63, MEH158 and MEH183 showed negligible haemolytic activities against rabbit erythrocytes and no cytotoxic activity *in vivo*. The toxic activity of MEH63, MEH158 and MEH183 was assessed *in vitro* (haemolysis assay) and *in vivo*. **(a)** The treatment of rabbit erythrocytes with MEH63, MEH158 and MEH183 (400 µg mL^‐1^) led to 0.9%, 0.7% and 0.7% haemolysis respectively. The incubation of rabbit erythrocytes with normal saline and 0.1% Triton X‐100 resulted in 0% and 100% haemolysis respectively. Data are representative of three independent experiments, and error bars correspond to the mean ± SEM. The histopathological evaluation of **(b)** kidneys and **(c)** liver of mice receiving 8 μg per gram of MEH63, MEH158 and MEH183 (alone and in combination) was conducted every 12 h for three days. Furthermore, the mice receiving normal saline or 20 μg per gram of vancomycin every 12 h for three days served as the controls. No toxic activity or tissue damage was observed in the kidneys or liver. Yellow arrowhead, renal corpuscle; red arrowhead, hepatocytes; red arrow, sinusoids; and V, central vein.

The haemolytic activity of the three scFvs was evaluated in rabbit erythrocytes. Based on the results, haemolysis by MEH63, MEH158 and MEH183 (at concentration of 400 µg mL^−1^) was less than 1% (0.9%, 0.7% and 0.7% respectively) compared with 0.1% Triton X‐100 (as 100% haemolysis) (Figure 5a). The potential toxic effects of anti‐*S*. *aureus* scFvs on the kidneys and liver of mice receiving MEH63, MEH158, MEH183 or a combination of all three scFvs were assessed histopathologically after 72 h and compared with the control groups, receiving vancomycin or normal saline. Following repeated dosing with anti‐*S*. *aureus* scFvs (8 µg per gram every 12 h), no acute toxicity, resulting in tissue damage, was found in the kidneys or liver of tested mice. Likewise, similar results were observed in the control groups (Figure 5b and c).

### Therapeutic efficacy of MEH63, MEH158 and MEH183 (alone and in combination) in a mouse model of bacteraemia

**Figure 6:**
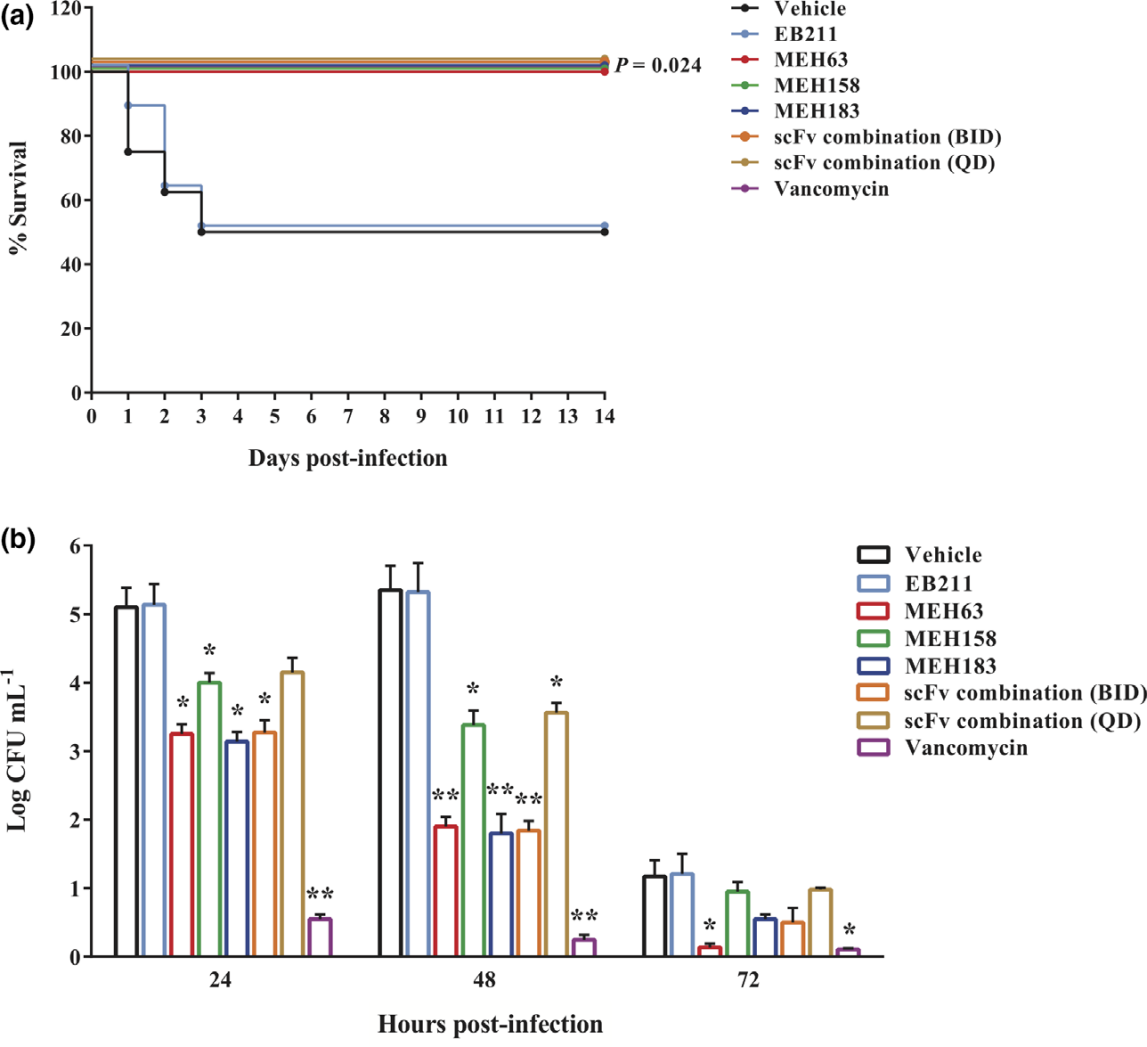
MEH63, MEH158 and MEH183 exhibited therapeutic efficacy in a mouse model of MRSA bacteraemia. To assess the therapeutic activity of MEH63, MEH158 and MEH183, the mice (n = 8) were intravenously infected with MRSA S.a.124 (~ 10^8^ CFU). Two h after the challenge, the infected mice were intraperitoneally treated with MEH63, MEH158, MEH183, the scFv combination, vancomycin, EB211 (an unrelated scFv antibody) or normal saline (vehicle) every 12 h (BID) for three days. The scFv combination was also administrated every 24 h (QD) for three days. **(a)** Mortality was recorded daily for two weeks, and the survival rate was calculated using the log‐rank test (Mantel–Cox test). Data are representative of three independent experiments. * *P*‐value = 0.024. **(b)** The bacterial colony‐forming unit count in the blood of infected mice (n = 6), treated with MEH63, MEH158, MEH183, vancomycin, EB211 or normal saline BID for three days. The scFv combination was also administrated QD for three days. Data are representative of three independent experiments, and error bars correspond to the mean ± SEM. * *P*‐value < 0.05, ** *P*‐value < 0. 01 and *** *P*‐value < 0.001.

The protective activities of MEH63, MEH158 and MEH183 (alone and in combination) were evaluated in a murine model of bacteraemia caused by MRSA S.a.124. After a 2‐week follow‐up, 100% of mice receiving MEH63, MEH158, or MEH183 at 2 h after the challenge survived (with normal cage activity and no hunched back or ruffled fur). In contrast, half of the mice in the vehicle group and the mice receiving EB211 (an anti‐*A. baumannii* scFv) succumbed to the infection (50% survival) (Figure 6a). Notably, all deaths in the two latter groups occurred on the first three days of the challenge. As illustrated in Figure 6a, the therapeutic administration of the combination of three scFvs (every 12 h and every 24 h for three days) and vancomycin caused a marked improvement in the survival rate of infected mice (100% survival).

The *S*. *aureus* load in the blood of infected mice, receiving the anti‐*S*. *aureus* scFvs (alone and in combination), vancomycin and EB211 2 h post‐infection, was compared with the vehicle group in the 72‐h post‐infection treatment (Figure 6b). Compared with the vehicle group, every 12‐h administration of MEH63, MEH183 and the scFv combination could significantly decrease the bacterial load in the blood of infected mice at 24 h post‐infection (Figure 6b). Also, vancomycin and EB211 exerted the highest and the lowest effects on the bacterial burden compared with the vehicle group (Figure 6b). Moreover, similar reducing effects were found on the bacterial burden in the blood of infected mice at 48 h post‐infection. Although the bacterial load in the blood decreased at 72 h post‐infection, MEH63 and vancomycin exhibited the greatest reducing activity (Figure 6b).

**Figure 7:**
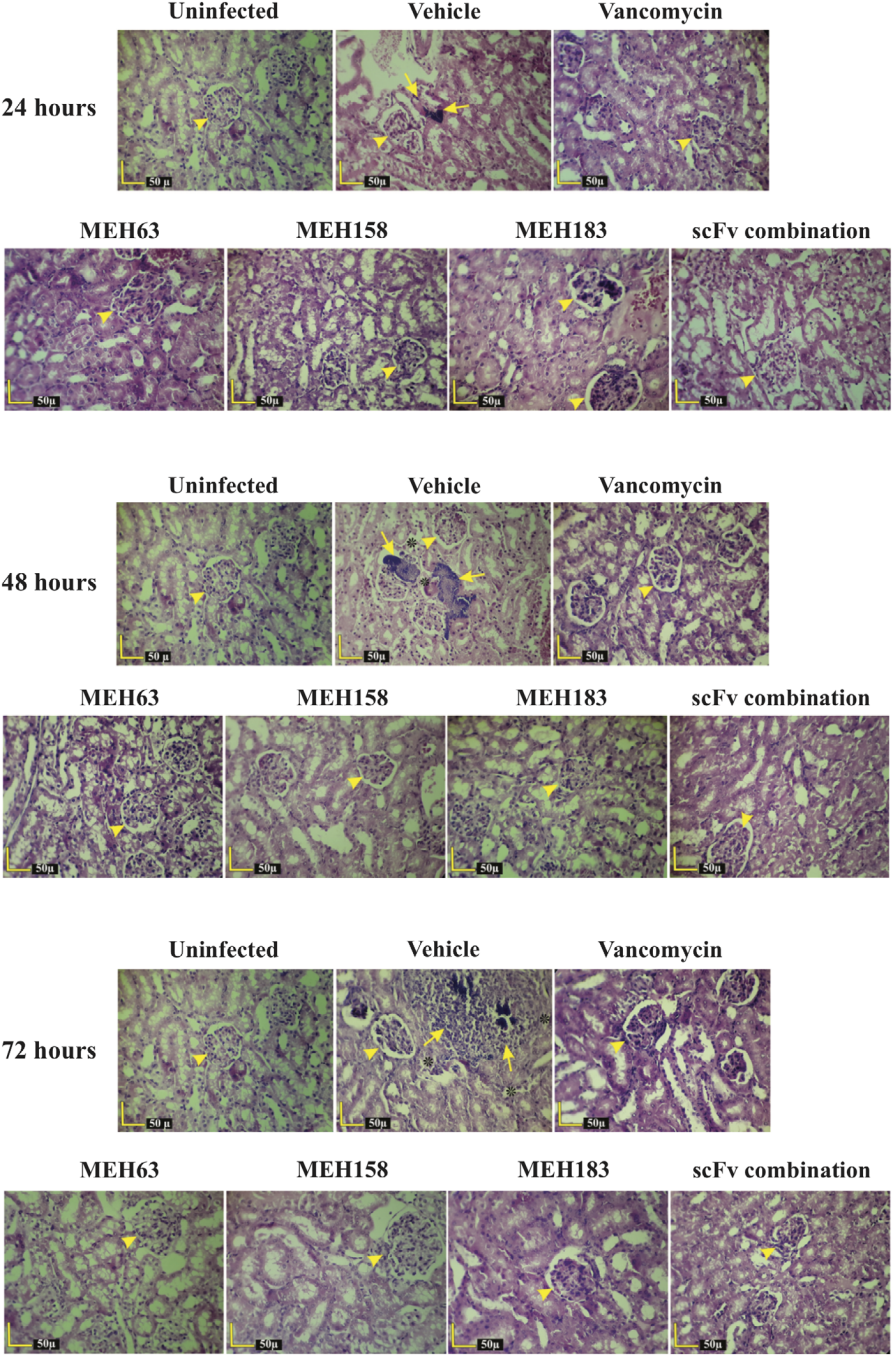
MEH63, MEH158 and MEH183 attenuated *S*. *aureus*‐induced inflammation and prevented tissue damage in kidneys. Mice (n = 6) were intravenously infected with MRSA S.a.124 (~ 10^8^ CFU). Two h after the challenge, the infected mice were intraperitoneally treated with MEH63, MEH158, MEH183, the scFv combination, vancomycin or normal saline (vehicle) every 12 h (BID) for three days. The kidneys were harvested from the uninfected mice, the vehicle group and infected mice receiving scFvs or vancomycin. The H & E‐stained sections of tissues were histopathologically examined. Bacterial foci and inflammatory infiltration of neutrophils and macrophages (yellow arrows) at 24, 48 and 72 h after the challenge, besides necrosis of the lining epithelium of renal tubules (asterisks) at 48 and 72 h after the challenge, are marked pathological events in the kidneys of the vehicle group, but not in the groups treated with either anti‐*S*. *aureus* scFvs or vancomycin. Yellow arrowhead, renal corpuscle.

**Figure 8:**
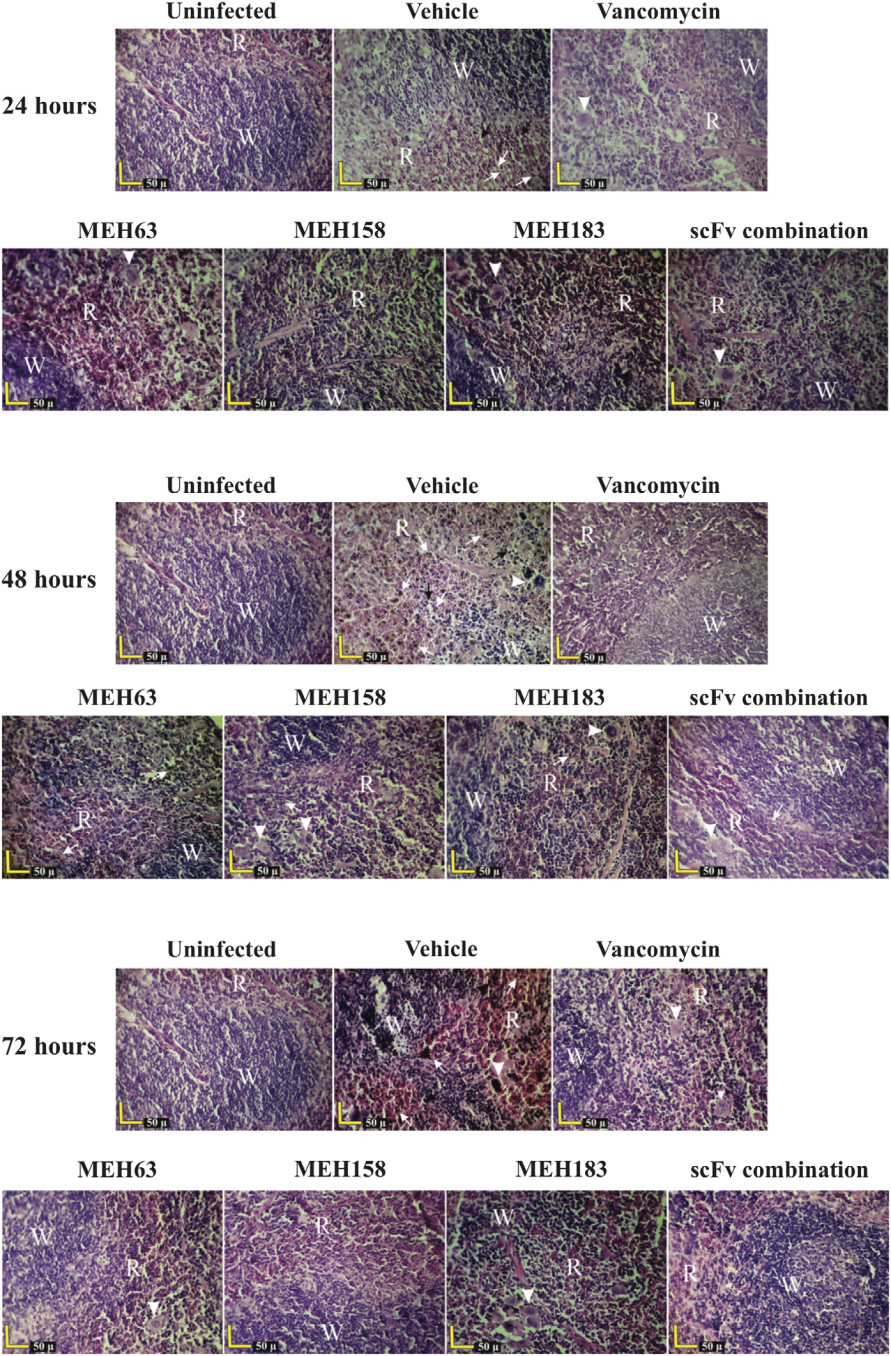
MEH63, MEH158 and MEH183 attenuated *S*. *aureus*‐induced inflammation and prevented tissue damage in the spleen. Mice (n = 6) were intravenously infected with MRSA S.a.124 (~ 10^8^ CFU). Two h after the challenge, the infected mice were intraperitoneally treated with MEH63, MEH158, MEH183, the scFv combination, vancomycin or normal saline (vehicle) every 12 h (BID) for three days. The spleens were harvested from the uninfected mice, the vehicle group and infected mice receiving scFvs or vancomycin. The H & E‐stained sections of tissues were histopathologically examined. Basophilic granular materials and macrophages (white arrows) and widened sinusoids (black arrows) were observed after 24, 48 and 72 h of infection in the spleen of mice in the vehicle group. Also, the excessive accumulation and congestion of blood (black arrowheads) and hyperplastic Malpighian follicles with fragmented cells were observed after 48 and 72 h of infection in the vehicle group. At 24, 48 and 72 h after infection, no significant pathological symptoms were found in mice treated with MEH63, MEH158, MEH183, the scFv combination or vancomycin. White arrowhead, megakaryocyte; R, red pulp; and W, white pulp.

The effectiveness of MEH63, MEH158 and MEH183 was further demonstrated by the histopathological assessment of the kidneys and spleen of infected mice treated with the anti‐*S*. *aureus* scFvs at 2 h post‐challenge (Figures 7 and 8). Besides bacterial communities with infiltration of neutrophils and macrophages, which were frequently observed in the kidneys of the vehicle group at 24, 48 and 72 h post‐challenge, necrosis of the lining epithelium of renal tubules was also detected at 48 and 72 h post‐infection, which might result from the exacerbation of infection (Figure 7). On the contrary, no bacterial foci, inflammation or tissue damage in the kidneys, resulting from infection with *S*. *aureus,* was found in mice treated with MEH63, MEH158, MEH183, the scFv combination or vancomycin (Figure 7).

In addition to basophilic granular materials and a large number of macrophages in the spleen of the vehicle group at 24, 48 and 72 h post‐challenge, excessive accumulation and congestion of blood and hyperplastic Malpighian follicles with fragmented cells were the most significant pathological symptoms in this group at 72 h post‐challenge (Figure 8). Notably, no marked histopathological alterations were found in the spleen of mice receiving MEH63, MEH158, MEH183, the scFv combination or vancomycin at 24, 48 and 72 h post‐challenge (Figure 8). Therefore, the therapeutic administration of anti‐*S*. *aureus* scFvs could lead to the inhibition of MRSA and prevent inflammation and tissue damage resulting from infection with MRSA.

## Discussion

The cell wall of bacteria with multiple virulence factors, which enables them to colonise in the host, evade the immune system or invade the host cell, has been one of the most critical targets for the design and development of antibacterial agents.^3^, ^5^, ^15^, ^45^, ^46^, ^47^, ^48^ In recent decades, significant attention has been paid to the antibody‐based immunotherapy of patients with infectious diseases either to decrease the consumption of antibiotics or to boost the therapeutic efficiency of conventional antibiotics for reducing the prevalence of resistant strains.^15^, ^49^ However, up to now, the U.S. Food and Drug Administration has only approved three mAbs, including raxibacumab and obiltoxaximab (against the anthrax toxin of *Bacillus anthracis*) and bezlotoxumab (against the toxin B of *Clostridium difficile*), which bind to and neutralise toxins.^50^

As the cell wall of *S. aureus* is composed of various virulence factors, some of which show redundancy,^51^ targeting multiple sites of this intricate structure with antibody fragments, such as scFvs, with great binding potential, small size and high tissue penetration may be effective.^28^, ^29^ Therefore, to identify scFv antibodies with potential binding to multiple factors involved in the survival, colonisation, evasion and invasion of *S. aureus*, a fully human scFv phage library was enriched against live MRSA strains grown with human PBMCs and human whole blood, or as a biofilm. Following the screening, six scFv antibodies were isolated, three of which (MEH63, MEH158 and MEH183) were selected for further evaluation, considering their acceptable expression yields and unique sequences.

As the function of an antibody is weighed by its ability to recognise and bind to the antigen, the binding ability of MEH63, MEH158 and MEH183 to *S. aureus* was assessed, and the results demonstrated the great potential of all three scFv antibodies in the identification of *S. aureus*. To determine whether the binding of scFvs to *S. aureus* was followed by growth inhibition or not, four *S. aureus* strains (*S. aureus* S.a.48, S.a.61, S.a.124 and ATCC 6538) were treated with the scFvs (MEH63, MEH158 and MEH183), leading to the markedly decreased viability of *S. aureus* compared with the control group. Consistent with our findings, several previous studies have reported the significant bactericidal activity of scFv fragments,^33^, ^34^, ^35^, ^36^ including ZWs, CB515 and S20, against *S. aureus* ^36^, relapsing fever *Borrelia* ^33^ and *Pseudomonas aeruginosa* serotype O6^34^, ^35^ respectively. The ZW scFvs were isolated from an scFv phage‐display library, constructed from PBMCs of cows with mastitis.^36^ Among eight ZWs (ZW1, ZW2, ZW12, ZW22, ZW33, ZW68, ZW73 and ZW88), showing growth inhibitory activities against *S*. *aureus* in an *in vitro* assay, ZW12, ZW88 and combination of the eight scFvs significantly inhibited the growth of bacteria. Based on the *in vitro* results, the prophylactic activity of the combination of eight scFvs was assessed in mice with *S*. *aureus* mastitis, indicating the significant protection of infected mice. Although the exact mechanism of the inhibitory activity of the ZW scFvs has not been determined, we can hypothesise that the eight scFvs (alone and in combination) affect the biological activity of the bacterium by binding to the cell surface of *S*. *aureus* (e.g. prevention of adhesion to host cells).^36^ The CB515 scFv, derived from an antivariable small protein (Vsp) IgM (CB515), exhibited bactericidal activity against relapsing fever *Borrelia* in a complement‐independent manner.^33^ This scFv displayed specific binding to Vsp (species‐ and serotype‐specific) and exerted its bactericidal effect in a dose‐dependent way, resulting in severe damage to the spirochetal outer membrane.^33^ LaRocca *et al*. attributed this bactericidal activity to the variable domains of the CB515 IgM.^33^ Moreover, the S20 was a bactericidal scFv, derived from a tobacco‐expressed human S20 IgG1, targeting LPS of *P. aeruginosa* (serotype O6ad).^35^ It considerably reduced the cell viability of *P. aeruginosa* O6ad *in vitro* and conferred protection in an *in vivo* model of *P. aeruginosa* infection.^35^ In another study, Richard *et al*. confirmed that the S20 scFv exerted its antibacterial activity through binding to the O‐specific antigen of *P. aeruginosa* (serotype O6), leading to the outer membrane disruption, followed by bacterial death.^34^ The direct bactericidal activity of scFv fragments can be explained by their function as AMPs^35^, ^37^, ^38^ or catalysis of some chemical reactions (e.g. abzymes),^35^ besides interference with the biological function of the bacterium as mentioned above.^35^, ^39^ We hypothesised that the binding of three scFvs (MEH63, MEH158 and MEH183) to *S. aureus* proteins might disrupt the function of bacteria, leading to bacterial death. In this regard, three *S. aureus* proteins, including TrkH family potassium uptake protein, PgeF and lpl8, which contributed to the growth, resistance and invasiveness of pathogens, were found by epitope mapping and confirmed by the Western blot analysis. The TrkH family potassium uptake protein, recognised by MEH63, is the potassium‐conducting subunit of the Trk transport system, involved in the vitality of bacteria.^52^, ^53^, ^54^, ^55^ In this regard, Rhoads *et al*. showed that *E. coli* bacteria with an inactivating mutation in the potassium uptake systems (Kup, Kdp and Trk) could not grow in potassium‐limited media.^55^ To determine the role of TrkH in bacterial virulence, Alkhuder *et al*. reported that *Francisella tularensis* with a defective *trkH* gene exclusively grew in high potassium‐containing media, affecting its survival and infectivity *in vivo*.^56^ Moreover, several studies have reported the role of two candidate proteins, PgeF and lpl8 (recognised by MEH158 and MEH183), in the vitality and pathogenicity of some pathogens.^57^, ^58^, ^59^, ^60^ PgeF (YfiH) is a less‐studied protein, preventing variations in the peptide chain composition of peptidoglycan in some bacteria.^57^, ^58^ Parveen *et al*. demonstrated that the *E. coli* mutant, devoid of the *yfiH* gene, generated defective peptidoglycan, making it more sensitive to β‐lactams.^57^ The last protein, lpl8, which belongs to the lipoprotein family, not only plays a significant role in the survival of *S. aureus* (as transporters or enzymes) but also is involved in the host–pathogen interactions (e.g. recognition by Toll‐like receptor 1 [TLR1]‐TLR2 or TLR2‐TLR6).^59^, ^61^ The recognition of lpls by the innate immune system resembles a double‐edged sword; although it triggers defence against the pathogen, it provides better conditions for *S. aureus* to invade host cells and disseminate further in the host.^61^ Furthermore, lpls have remarkable features, such as a conserved core domain and a protein part accessible to antibodies; therefore, they can be suitable vaccine candidates or functional targets for the generation of therapeutic antibodies.^59^ It should be noted that MEH63, MEH158 and MEH183 lost their antibacterial activity in the presence of high concentrations of Mg^2+^. The frequency of basic residues (arginine and lysine) in MEH63, MEH158 and MEH183 (pI > 9) enabled these positively charged scFvs to interact with the negatively charged components of the cell wall and displace divalent cations, such as Mg^2+^.^43^ The electrostatic interaction between cationic antimicrobial agents and teichoic acids can be interrupted at a high concentration of Mg^2+^, resulting in the loss or reduction of bactericidal activity against *S. aureus*.^41^, ^62^, ^63^, ^64^, ^65^, ^66^ Therefore, we hypothesised that anti‐*S*. *aureus* scFvs showed bactericidal activity against *S. aureus* by disturbing the cell wall integrity and binding to proteins involved in the survival of *S. aureus*.

The development of mAbs with broad bactericidal activities may help prevent or treat infections caused by multiple human pathogens. Besides *S. aureus*, *S. epidermidis* and *S. pyogenes* are two other life‐threatening Gram‐positive pathogens.^67^, ^68^, ^69^, ^70^ The former pathogen, which develops robust biofilms, is one of the major causes of device‐related infections,^67^ while the latter is responsible for a range of severe infections, including acute glomerulonephritis, rheumatic fever and toxic shock syndrome.^68^, ^69^, ^70^ In this study, we assessed the binding ability and growth inhibitory activity of MEH63, MEH158 and MEH183 against *S. epidermidis* and *S. pyogenes*. The results showed that all three scFvs could bind to *S. epidermidis* and *S. pyogenes* and affect their growth. Similar to the antibacterial activity of MEH63, MEH158 and MEH183 against *S. aureus*, the antibacterial activity against *S. epidermidis* and *S. pyogenes* was diminished in the presence of high concentrations of Mg^2+^. Considering the similar cell wall structure of Gram‐positive bacteria, it can be concluded that cationic scFvs might compromise the cell wall integrity of *S. epidermidis* and *S*. *pyogenes,* leading to their growth inhibition. Also, the antibacterial activities of MEH63, MEH158 and MEH183 might partly result from the binding of MEH63 to ABC transporter substrate‐binding proteins in *S. epidermidis* and *S*. *pyogenes*, besides the binding of MEH158 and MEH183 to the PgeF of *S. epidermidis* and D‐alanyl‐D‐alanine carboxypeptidase of *S*. *pyogenes*. ABC transporter substrate‐binding proteins play a significant role in the survival of bacteria (e.g. transportation of amino acids, vitamins and metal ions).^71^, ^72^, ^73^, ^74^, ^75^ Additionally, PgeF is needed to maintain the integrity of peptidoglycan,^57^ and D‐alanyl‐D‐alanine carboxypeptidase is involved in the maturation of peptidoglycan.^74^, ^75^, ^76^, ^77^ Therefore, MEH63, MEH158 and MEH183 can cause detrimental effects on bacterial growth by binding to these critical proteins and disrupting their activity. As a result, the growth inhibitory effects of MEH63, MEH158 and MEH183 on *S. epidermidis* and *S. pyogenes* may shed light on applying the scFvs in combination with antibiotics against infections caused by these pathogens.

Apart from the tremendous therapeutic efficacy of mAbs against various disorders (e.g. cancers, autoimmune disorders and infectious diseases), severe side effects, such as cardiotoxicity, anaemia, leucopenia, thrombocytopenia and severe hypersensitivity reactions, have restricted their broad applications.^78^, ^79^ As most of these unwanted side effects result from either the off‐target effects or mechanisms of action of mAbs, the potential binding of MEH63, MEH158 and MEH183 to human PBMCs and their haemolytic activity against rabbit erythrocytes were assessed. The results revealed that the scFvs had no binding ability to human PBMCs and lacked haemolytic activity. Consistent with the *in vitro* data, MEH63, MEH158 and MEH183 exhibited no off‐target activity or toxicity in the kidneys or liver of mice receiving the anti‐*S*. *aureus* scFvs (alone or in combination) at a dose of approximately 320 μg per day. According to these findings, the scFvs can recognise target proteins, which are not mutual with human proteins, and avoid the undesirable effects as the leading causes of early termination of clinical trials.

*Staphylococcus aureus* is armed with different virulent determinants. A promising therapeutic approach can involve targeting multiple factors to prevent the emergence of resistant strains and kill the bacterium exclusively.^3^, ^80^, ^81^ Ideally, the combination of MEH63, MEH158 and MEH183 at their lowest concentrations showed an almost similar bactericidal activity to MEH63, MEH158 and MEH183 at their highest concentrations, which might be associated with the combined effect of these three scFvs in compromising the cell wall integrity, besides concurrent targeting of the TrkH family potassium uptake protein and PgeF. Different combinations of mAbs, targeting various virulence factors of pathogens such as *S. aureus* ^82^ and *P. aeruginosa*,^49^ have been developed and evaluated. Tkaczyk *et al*. reported that concurrent targeting of alpha toxin and ClfA with two mAbs (MEDI4893* and 11H10 respectively) led to the prevention of alpha toxin‐related effects; inhibition of bacterial colonisation and dissemination to the bloodstream; broad coverage of strains; enhancement of opsonophagocytosis; and significant prophylactic effects compared with individual mAbs in a mouse model of *S. aureus* bacteraemia.^82^ Furthermore, in a study by DiGiandomenico *et al*., the prophylactic administration of two mAbs (targeting the type III secretion protein PcrV and exopolysaccharide Psl), with diverse roles in *P. aeruginosa*‐associated infections, provided greater protection against *P. aeruginosa* strain 6206, compared with each mAb alone, in a mouse model of lethal pneumonia.^49^ Therefore, the concurrent use of MEH63, MEH158 and MEH183 can prevent the emergence of resistant bacteria since mutations altering the cell wall and target proteins, such as TrkH, PgeF and lpl8, might significantly affect the viability and pathogenesis of bacteria.

Besides the emergence of vancomycin‐resistant strains, adverse events, such as thrombocytopenia,^83^ autotoxicity, neutropenia, fever, red man syndrome^84^ and nephrotoxicity,^85^ highlight the need for an effective treatment regimen for patients with MRSA infections.^86^, ^87^, ^88^ These limitations might be overcome by combining the classic antibiotics at lower concentrations with novel therapeutics,^87^, ^88^ such as antibodies. In the present study, the combination of vancomycin and MEH63, MEH158 and MEH183 produced additive effects. Consistent with our findings, Mohamed *et al*. reported the additive effect of two antimicrobial peptides (designated RRIKA and RR) and vancomycin against MSSA, MRSA, vancomycin‐intermediate *S. aureus*, vancomycin‐resistant *S. aureus* and *S. epidermidis*.^89^ The additive effect revealed that the antibiotic and the scFvs functioned independently and exerted their bactericidal effects by targeting different sites of *S. aureus*.^42^ Moreover, MEH63, MEH158 and MEH183, by compromising the cell wall integrity, presumably accelerated the penetration of vancomycin.^86^, ^90^

*Staphylococcus aureus*‐associated bacteraemia, with a high incidence rate, inadequate clinical response and mortality rate of approximately 20%, has been one of the most challenging problems in the healthcare systems.^7^, ^91^ Therefore, we appraised the therapeutic efficacy of MEH63, MEH158 and MEH183 (alone and in combination) in a mouse bacteraemia model. The results showed diminished bacterial burden in the blood, decreased tissue damage and inflammation and prolonged survival in scFv‐treated mice (100% survival) compared with the control groups, which either did not receive treatment or received an unrelated scFv fragment. In this regard, Ohsawa *et al*. showed the therapeutic efficacy of a mouse anti‐peptidoglycan mAb (ZBIA5H) in a mouse model of sepsis, induced by either community‐acquired MRSA or vancomycin‐resistant *S. aureus* strains (survival rate ≥ 50).^92^ In another study, a human mAb targeting the immunodominant staphylococcal antigen A (1D9) demonstrated only prophylactic activity (not therapeutic activity) and improved survival in a mouse model of bacteraemia, induced by the *S. aureus* isolate P (an MSSA strain).^25^ However, it did not exhibit any prophylactic or therapeutic effects in mice challenged with the *S. aureus* USA300 (an MRSA strain).^25^ In this regard, Van den Berg *et al*. suggested that the latter might result from the single‐dose administration of 1D9 at 3 h post‐infection, leading to the low accessibility of mAb to bacteria disseminated to various tissues.^25^ Also, to assess the protective efficacy of the S20 scFv (an anti‐*P. aeruginosa* antibody), the leucopenic mice, challenged with the *P. aeruginosa* O6ad strain, received the scFv prophylactically, resulting in their prolonged survival compared with the control groups without treatment.^35^ In contrast to previous studies, MEH63, MEH158 and MEH183 were administrated every 12 h for three days, leading to considerably prolonged survival in the treated mice compared with the control groups. The results also indicated the potential of these scFvs as therapeutic agents for treating *S*. *aureus* bacteraemia.

In conclusion, MEH63, MEH158 and MEH183 could inhibit the growth of *S*. *aureus* and confer protection against the MRSA challenge in a mouse model of bacteraemia. Based on the results, direct bactericidal activities of MEH63, MEH158 and MEH183 resulted from the cell wall interruption, besides targeting proteins involved in the survival and pathogenicity of *S*. *aureus*. These scFv antibodies, which directly destroy the pathogens without immune system members’ assistance,^33^, ^35^, ^39^, ^93^, ^94^, ^95^, ^96^, ^97^, ^98^ can provide a promising treatment option for patients with immune system disorders. Considering the lack of off‐target activity on human cells and the additive effects with vancomycin, these bactericidal scFvs (alone or in combination) can be used along with other antibiotics as encouraging therapeutics to diminish the challenges of treatment in patients with bacteraemia.

## Methods

### Bacteria and growth conditions

Three MRSA strains (*S. aureus* S.a.48, isolated from the cerebrospinal fluid; *S. aureus* S.a.61, isolated from an intravascular catheter; and *S. aureus* S.a.124, isolated from the blood culture of a patient) were provided by the Department of Mycobacteriology and Pulmonary Researches of Pasteur Institute of Iran.^99^ An extensively drug‐resistant clinical isolate of *A. baumannii* (A.b.56) was obtained from the Microbiology Laboratory of Pasteur Institute of Iran.^100^ Also, *S. aureus* ATCC 6538, *S*. *epidermidis* ATCC 12228 and *S*. *pyogenes* ATCC 10403 were obtained from the American Type Culture Collection. All strains were grown in trypticase soy broth (TSB; Sigma‐Aldrich, Saint Louis, USA) and trypticase soy agar (TSA; Sigma‐Aldrich) or blood agar (TSA enriched with 5% sheep blood; Darvash Co., Tehran, Iran). The bacterial titres were determined based on the OD_600_ and confirmed by plating on TSA plates. The incubation temperature was 37°C for all strains.

### Determination of the MICs of oxacillin and vancomycin

The MICs of oxacillin against *S. aureus* S.a.48, S.a.61, S.a.124 and ATCC 6538 were measured by the MIC test strip (0.0625–32 µg mL^−1^) (Liofilchem Co., Roseto, Italy), according to the manufacturer’s instructions. Moreover, the MICs of vancomycin for *S. aureus* S.a.48, S.a.61, S.a.124 and ATCC 6538, and *S. epidermidis* ATCC 12228 and ampicillin for *S. pyogenes* ATCC 10403 were measured, using the broth microdilution method, according to the guidelines of the Clinical and Laboratory Standards Institute (CLSI).^101^

### Biofilm assay

The biofilm formation ability of four strains (*S. aureus* S.a.48, S.a.61, S.a.124 and ATCC 6538) was determined after 24 and 72 h by the crystal violet staining assay as described previously by Irani *et al*.^100^ The mean OD of the negative control (the well containing the uninoculated medium) plus three standard deviations (SDs) was defined as the cut‐off OD value. The strains were classified into non‐biofilm producers and weak, moderate and strong biofilm producers based on the results.^100^

### Enrichment of a fully human scFv library against living *S. aureus*

A phage‐display human scFv library, with total diversity of 2 × 10^10^,^102^, ^103^, ^104^, ^105^ was screened against two MRSA strains grown in human PBMCs with plasma (PBMC–plasma biopanning), in human whole blood (blood biopanning)^100^ or as a biofilm (biofilm biopanning).^100^ In brief, an overnight culture of bacteria (*S. aureus* S.a.48 and S.a.124), grown in the medium containing TSB and PBMCs plus plasma (or human whole blood), was diluted 1:50 in fresh medium and incubated for 3 h at 37°C. Next, the bacteria were centrifuged at 2000 *g* for 10 min and resuspended in 1 mL of phosphate‐buffered saline (PBS). For the biofilm biopanning, the biofilm produced by the bacteria (*S. aureus* S.a.61 and S.a.124) in a 96‐well flat‐bottom microplate after 72 h was used for enrichment.^100^ In parallel, the fully human scFv phage library was amplified, and biopanning was carried out as described previously.^100^ Briefly, the purified phages (~10^12^ CFU mL^−1^) were incubated for 1 h at room temperature (RT) with bacteria (~10^8^ CFU mL^−1^) grown in the three media described above. After washing with PBS plus 0.5% Tween‐20 (PBST) three times, the phages binding to the bacteria were eluted and amplified for the next round of biopanning. This process was repeated for four rounds, and the washing steps were increased from three to seven times to isolate more specific phages. In each round of biopanning, the titres of the inputs and outputs were determined, and the input/output ratio was measured to evaluate the enrichment efficiency.^100^

### Assessment of phage binding to *S. aureus*

To evaluate the binding ability of the purified phages from output_1_–output_4_ of PMBC–plasma, blood and biofilm biopanning to MRSA strains, the dot‐blot assay was carried out (polyclonal phage assay).^100^ Briefly, 20 µL of bacterial suspensions diluted in PBS (~10^8^ CFU mL^−1^) was spotted on the nitrocellulose membranes (GE Healthcare, Little Chalfont, UK). After blocking, the membranes were individually incubated with the phages (output_1_–output_4_) or helper phages (Thermo Scientific, MA, USA) for 1 h at RT. After several washing steps with tris‐buffered saline (TBS), containing 0.05% Tween‐20 (TBST), and incubation with horseradish peroxidase (HRP)‐conjugated anti‐M13 major coat protein antibody (1:2000 dilution) (Santa Cruz Biotechnology Inc., Heidelberg, Germany) for 1 h, the reactions were developed by adding diaminobenzidine (DAB) (Sigma‐Aldrich) and hydrogen peroxide (H_2_O_2_), according to the manufacturer’s instructions.

To determine monoclonal phages with the best binding ability to the MRSA bacteria (monoclonal phage assay), the phages from the third round of PMBC–plasma, blood and biofilm biopanning (output_3_), which showed significant reactions relative to the other rounds (output_1_, output_2_ and output_4_) and the controls, were used to infect exponentially growing *E*. *coli* TG1. The infected cells were cultured on Lysogeny broth (LB) agar (Merck, Darmstadt, Germany) plates containing ampicillin (100 µg mL^−1^), and the plates were incubated overnight at 37°C. The grown colonies were randomly selected, and the purified phages were assessed for their binding to MRSA bacteria, using the dot‐blot assay as described earlier.

### Expression of soluble scFv antibodies, SDS‐PAGE and Western blot analysis

To produce soluble scFv antibodies, exponentially growing *E. coli* HB2151 bacteria were infected with phages purified from 11 phage clones (MEH63, MEH79 and MEH94 from PBMC–plasma biopanning; MEH121, MEH131, MEH169 and MEH178 from blood biopanning; and MEH158, MEH183, MEH188 and MEH199 from biofilm biopanning), exhibiting significant binding in the monoclonal phage assay.^104^ The expression of scFv antibodies was induced by isopropyl β‐d‐1‐thiogalactopyranoside (0.1 mM IPTG) (GE Healthcare) as previously described by Eisenhardt *et al*.^106^ Next, the presence and the expression level of scFv fragments in the periplasmic extracts of *E. coli* HB2151 bacteria, harbouring the selected phagemids (MEH63, MEH158, MEH169, MEH178, MEH183 and MEH188), were analysed by SDS‐PAGE and Western blotting. For Western blotting, the blocked polyvinylidene fluoride (PVDF) membrane (GE Healthcare), containing proteins transferred from the 12% SDS‐PAGE gel, was incubated with mouse anti‐human scFv polyclonal antibody (Supplementary figure 18) for 1 h at RT. After several washing steps with TBST and TBS and then incubation with goat anti‐mouse IgG‐HRP antibody (1:2000 dilution) (Santa Cruz) for 1 h at RT, the membrane was washed, and the signals were detected using DAB, according to the manufacturer’s instructions.

### Sequencing

The plasmids of six clones, MEH63, MEH158, MEH169, MEH178, MEH183 and MEH188, with high expression levels, were extracted using the High Pure Plasmid Isolation Kit (Roche, Mannheim, Germany), according to the manufacturer’s instructions. The forward primer, 5′‐CTA TCA GCA AGA TAA GCA AAT AGT T‐3′, was used for sequencing. The nucleotide sequences were assessed in Gene Runner program version 6.0, and then, the inferred amino acid sequences were examined by the IMGT V‐QUEST (http://www.imgt.org/IMGT_vquest/analysis).^107^

### Evaluation of the binding ability of the selected scFv antibodies to *S. aureus*

Among six scFv antibodies, MEH63, MEH158 and MEH183 were purified by IMAC using Ni‐NTA agarose (Qiagen, Hilden, Germany), according to the manufacturer’s instructions. The fractions were eluted with a buffer containing 200 mM imidazole. Next, the eluted fractions were dialysed against PBS, using a pre‐swollen dialysis bag with a cut‐off molecular weight of 14 kDa (Sigma‐Aldrich), based on the manufacturer’s instructions. The concentration and purity of scFv antibodies were assessed by the Bradford assay and SDS‐PAGE respectively.

The purified scFv antibodies’ ability to bind to MRSA bacteria was evaluated by the dot‐blot assay as described earlier. Briefly, bacteria (*S. aureus* S.a.124, *S. epidermidis* ATCC 12228, *S. pyogenes* ATCC 10403 and *A. baumannii* A.b.56, ~10^8^ CFU mL^−1^), spotted on the nitrocellulose membranes, were incubated individually with the purified scFv antibodies (MEH63, MEH158 or MEH183) or bovine serum albumin (BSA) (Merck) for 1 h at RT. Next, the membranes were washed and incubated with mouse anti‐human scFv antibody for 1 h at RT. After multiple washing steps, followed by incubation with goat anti‐mouse IgG‐HRP antibody for 1 h at RT, the membranes were washed, and the reactions were developed using DAB and H_2_O_2_, according to the manufacturer’s instructions. Also, to predict the off‐target potential of the selected scFv antibodies, 20 µL of human PBMCs, which was diluted in 1 mL of PBS (~10^7^ cells mL^−1^), was spotted on the nitrocellulose membranes and incubated individually with the scFv antibody (MEH63, MEH158 or MEH183). After incubation with mouse anti‐human scFv antibody for 1 h and then goat anti‐mouse IgG‐HRP antibody, the reactions were developed using DAB and H_2_O_2_.

### Assessment of the growth inhibitory effect of the selected scFv antibodies on *S. aureus*

The inhibitory effect of MEH63, MEH158 and MEH183 on the growth of *S. aureus* was assessed using the microtitre plate and agar plate assays as described previously with some modifications.^34^, ^35^, ^38^ Using the microtitre plate technique, 50 µL of bacterial suspensions (*S. aureus* S.a.48, S.a.61, S.a.124 and ATCC 6538, ~10^8^ CFU mL^−1^) was initially incubated with an equal volume of vancomycin (0.0625–32 µg mL^−1^) for 20 h at 37°C, and the OD_600_ was read every hour for 10 h and once after 20 h. After assessing the growth curves of four strains treated with vancomycin (positive control), 50 µL of bacterial suspensions of *S. aureus* S.a.48, S.a.61, S.a.124 and ATCC 6538 was individually incubated with an equal volume of MEH63, MEH158 and MEH183 (at a final concentration of 200 µg mL^−1^), as described for vancomycin, and their growth curves were compared with the growth curves of untreated bacteria (incubated with PBS), bacteria incubated with the denatured MEH158 (scFv heated at 100°C for 30 min) and bacteria incubated with vancomycin (1 µg mL^−1^ for *S. aureus* ATCC 6538 and 2 µg mL^−1^ for *S. aureus* S.a.48, S.a.61 and S.a.124 strains).

In the agar plate method, 50 µL of bacterial suspensions (*S. aureus* S.a.48, S.a.61, S.a.124 and ATCC 6538) was incubated with an equal volume of vancomycin (0.0625–32 µg mL^−1^), MEH63, MEH158 or MEH183 (at a final concentration of 200 µg mL^−1^) for 4 h at 37°C. Then, 10 µL of the 10‐fold diluted suspension was spread on the LB agar or the LB agar containing oxacillin. After 18 h of incubation at 37°C, the colonies were enumerated. Also, an equal volume of bacterial suspensions of *S. aureus* S.a.48 and S.a.124 was incubated individually with an equal volume of different concentrations of MEH63, MEH158 or MEH183 (25, 100 and 200 µg mL^−1^) and in combination with three scFv antibodies (at a final concentration of 200 µg mL^−1^) for 4 h at 37°C. After plating 10 µL of the diluted suspensions (scFv‐treated and untreated groups) on the LB agar containing oxacillin and incubation at 37°C for 18 h, the colonies were enumerated. The untreated bacteria (incubated with PBS) were considered as the control.

### Evaluation of the inhibitory activity of *S. aureus*‐specific scFvs on *S. epidermidis*, *S. pyogenes* and *A. baumannii*

The effects of MEH63, MEH158 and MEH183 on the growth of *S. epidermidis* ATCC 12228, *S. pyogenes* ATCC 10403 and *A. baumannii* A.b.56 were assessed using the agar plate method as described above. The untreated bacteria (incubated with PBS) were considered as the control.

### Epitope mapping

To identify epitopes recognised by MEH63, MEH158 and MEH183, a Ph.D.TM‐C7C Phage Display Peptide Library (New England Biolabs, Beverly, MA, USA) was screened against the scFv (MEH63, MEH158 or MEH183), according to the manufacturer’s instructions. Single‐stranded DNAs of 12 phage clones (four phage clones from the third round of biopanning on MEH63, MEH158 or MEH183) were extracted and sequenced, according to the Ph.D.‐C7C Kit instructions. The inference of amino acid sequences was analysed in Gene Runner version 6.0 and checked in the Biopanning Data Bank to remove probable target‐unrelated peptides (MimoDB) (http://immunet.cn/bdb/).^108^, ^109^ Next, the selected peptides were blasted against the NCBI protein database for *S. aureus* *,* *S. epidermidis* and *S. pyogenes* *,* and proteins with scores above 18 were selected as the candidate proteins.^108^, ^109^

### Western blot analysis

A crude cell wall extract of *S. aureus* was prepared as previously described.^110^, ^111^ An overnight culture of *S. aureus* S.a.124 was centrifuged at 4000 *g* for 15 min at 4°C. The pellet was resuspended in 1 mL of TBS, followed by disruption with glass beads (0.1 µm in diameter) using a Precellys 24 homogeniser (Bertin Technologies, Montigny‐le‐Bretonneux, France). After centrifugation, the supernatant was collected and centrifuged at 20 000 *g* for 15 min. Next, the pellet was resuspended in PBS and assessed by SDS‐PAGE. To extract non‐covalently bound cell wall proteins, the same overnight culture was centrifuged, and the pellet was washed with PBS, followed by incubation with potassium thiocyanate (1 M KSCN) for 10 min on ice. After centrifugation, the supernatant was precipitated with 10% trichloroacetic acid (TCA) for 10 min.^110^, ^111^ After centrifugation at 20 000 *g* for 5 min at 4°C, the pellet was washed several times with acetone, resuspended in loading buffer and evaluated by SDS‐PAGE.^110^, ^111^ The cell wall extracts of *S. epidermidis* ATCC 12228,^110^, ^111^ *S. pyogenes* ATCC 10403^112^ and *A. baumannii* A.b.56^113^, ^114^ were obtained as previously described.

To assess the binding of MEH63, MEH158 and MEH183 to *S. aureus* S.a.124*, S. epidermidis* ATCC 12228, *S. pyogenes* ATCC 10403 and *A. baumannii* A.b.56, the cell wall proteins, separated by SDS‐PAGE, were blotted onto the PVDF membranes. After blocking, the membranes were individually incubated with the scFv (MEH63, MEH158 or MEH183) for 1 h at RT. After incubation with mouse anti‐human scFv antibody and then goat anti‐mouse IgG‐HRP antibody, the bands were visualised with DAB and H_2_O_2_ (as described previously).

### Assessment of amino acid sequences of anti‐*S. aureus* scFvs

The amino acid sequences of the CDRs of MEH63, MEH158 and MEH183 were investigated in the APD (http://aps.unmc.edu/AP/main.php).^38^ Additionally, the physicochemical parameters of scFvs (e.g. pI and GRAVY scores of CDRs) were determined by the ExPASy’s ProtParam tool (https://web.expasy.org/protparam/).^115^

### Evaluation of the effect of Mg^+2^ on the bactericidal activity of anti‐*S. aureus* scFvs

High concentrations of divalent cations, such as Mg^2+^, can prevent the interactions between cationic antimicrobial agents and negatively charged cell wall components, leading to the decreased bactericidal activity.^41^, ^62^, ^63^, ^64^, ^65^, ^66^ The growth of *S. aureus* S.a.48, S.a.61, S.a.124 and ATCC 6538, *S. epidermidis* ATCC 12228 and *S. pyogenes* ATCC 10403 treated with MEH63, MEH158 and MEH183 (200 µg mL^−1^) in the presence of MgSO_4_ (5 and 20 mM)^41^, ^62^, ^66^ was assessed using the microtitre plate technique as described earlier.^34^, ^35^, ^38^, ^89^ Based on the agar plate technique, the bacterial suspension (*S. aureus* S.a.48, S.a.61, S.a.124 and ATCC 6538, *S. epidermidis* ATCC 12228 and *S. pyogenes* ATCC 10403) was mixed with an equal volume of MEH63, MEH158, MEH183 or vancomycin in the presence of MgSO_4_ (5 and 20 mM) at 37°C for 4 h. At 30 min and 4 h of incubation, 10 µL of diluted suspensions was spread onto the LB agar or the LB agar containing oxacillin. The bacterial colonies were counted after 18 h of incubation at 37°C. Besides, the control groups included bacteria incubated with PBS in the presence and absence of MgSO_4_ and bacteria incubated with the scFv or antibiotic (vancomycin for *S. aureus* and *S. epidermidis*, and ampicillin for *S. pyogenes*) in the absence of MgSO_4_.

### Checkerboard assay

The interactions between anti‐*S. aureus* scFvs (MEH63, MEH158 and MEH183) and vancomycin were assessed using the microdilution checkerboard assay.^116^ Briefly, inocula of 10^5^ CFU mL^−1^ were prepared for *S. aureus* S.a.48 and S.a.124. The scFvs (MEH63, MEH158, MEH183 and combination of three scFvs) and vancomycin were examined at concentrations of 3.125–200 µg mL^−1^ and 0.0625–16 µg mL^−1^ respectively. Bacterial growth was assessed after 18 h of incubation at 37°C. The wells containing incubated bacteria with PBS and uninoculated media were used as the controls. The activity of different combinations of scFvs and vancomycin was assessed by the fractional inhibitory concentration index (FICI), based on the following formula:

$$\text{FICI}={\text{FIC}}_{\text{scFv}}+{\text{FIC}}_{\text{Van}}$$

where FIC_scFv_ is the MIC of scFv in combination divided by the MIC of scFv alone, and FIC_Van_ is the MIC of vancomycin in combination divided by the MIC of vancomycin alone.

Based on the FICI values, the anti‐*S*. *aureus* scFv and antibiotic combinations were categorised into four groups: synergy (FICI ≤ 0.5), additive (0.5 < FICI ≤ 2), indifference (2 < FICI ≤ 4) and antagonism (FICI > 4).^117^

### Evaluation of the haemolytic activity of the anti‐*S. aureus* scFvs

The haemolytic potential of MEH63, MEH158 and MEH183 was assessed as previously described.^9^, ^100^, ^118^ Briefly, 100 µL of a 5% suspension of rabbit erythrocytes was incubated with an equal volume of the scFv (400 µg mL^−1^) for 1 h at 37°C. The suspensions incubated with normal saline and 0.1% Triton X‐100 were considered as the controls. After centrifugation, the supernatants were transferred to a new 96‐well plate, and the haemoglobin release was determined at OD_450_ nm, using a microtiter plate reader (BioTek, VT, USA). The percentage of haemolysis was calculated by the following formula:^9^

$$\%\text{Haemolysis}=\frac{\text{ODs}‐\text{ODn}}{\text{ODT}‐\text{ODn}}\times 100$$

OD_S_ is the absorbance of the scFv, OD_n_ is the absorbance of normal saline, and OD_T_ is the absorbance of 0.1% Triton X‐100.

### *In vivo* examination of cytotoxic effects of anti‐*S. aureus* scFvs

Female BALB/c mice (six per group, 6–8 weeks old), supplied by the Animal Laboratory of Pasteur Institute of Iran, intraperitoneally received 8 μg per gram of MEH63, MEH158, MEH183 or the scFv combination every 12 h for three days. The mice receiving normal saline or 20 μg per gram of vancomycin (every 12 h for three days) were considered the controls. To investigate histopathological alterations, all mice were sacrificed after 72 h, and the kidney and liver samples, stained with haematoxylin and eosin (H & E), were examined by light microscopy.^49^, ^119^, ^120^ All animal experiments were performed in accordance with the Animal Care and Use Committee guidelines of Pasteur Institute of Iran (IR.PII.REC.1394.23).

### Assessment of the therapeutic efficacy of MEH63, MEH158 and MEH183 in mice with *S. aureus* bacteraemia

To establish bacteraemia, female BALB/c mice (eight per group, 6–8 weeks old), supplied by the Animal Laboratory of Pasteur Institute of Iran, were intravenously injected with a lethal dose of 50% (LD_50_) of *S. aureus* S.a.124 (~10^8^ CFU per mouse).^120^, ^121^, ^122^ Two h after the bacterial challenge, the mice were intraperitoneally administrated with 8 μg per gram of MEH63, MEH158 or MEH183 every 12 h for three days. To determine whether the dosing regimen influenced the efficacy of scFvs, two groups were considered. One group received the combination of three scFvs (8 μg per gram) every 12 h, and the other group received the scFv combination (8 μg per gram) every 24 h for three days. The infected mice receiving 20 μg per gram of vancomycin,^92^ 8 μg per gram of EB211 (an unrelated scFv antibody targeting *A. baumannii*) and normal saline (vehicle group) every 12 h for three days served as the control groups. The clinical signs of mice were monitored for two weeks (mice with drastic signs of disease were euthanised), and the survival rate was calculated using the log‐rank test (Mantel–Cox test).^19^, ^26^, ^120^, ^121^, ^123^ To determine the effect of anti‐*S. aureus* scFvs on the bacterial burden in blood, mice (six per group), treated with MEH63, MEH158, MEH183, the combination of three scFvs, vancomycin, EB211 or normal saline 2 h post the challenge, were bled at 24, 48 and 72 h after the challenge. The blood samples, which were 10‐fold diluted with PBS serially, were cultured on the LB agar with oxacillin, and the CFU was enumerated after 18 h of incubation at 37°C.^120^

Moreover, the efficacy of MEH63, MEH158 and MEH183 was assessed based on the histopathological examination of the kidneys and spleen of mice receiving different treatments 2 h post‐infection. In this regard, the infected mice (six per group), which were administrated the anti‐*S*. *aureus* scFvs (alone and in combination), vancomycin, normal saline or EB211 (every 12 h for three days), were euthanised 24, 48 and 72 h after the challenge, and their kidneys and spleen were removed. The mice only receiving normal saline also served as the control (uninfected). The tissue sections were embedded in paraffin and stained with H & E, followed by examination under a light microscope to evaluate the histopathological alterations.^120^, ^122^, ^124^

### Statistical analysis

A Student’s *t*‐test was performed in GraphPad Prism version 6.07 for statistical analysis. *P*‐values less than 0.05 were considered statistically significant.

## Conflict of interest

The authors declare that they have no conflict of interest.

## Author contribution

**Behnoush Soltanmohammadi:** Data curation; Formal analysis; Investigation; Methodology; Software; Writing‐original draft; Writing‐review & editing. **Somayeh Piri‐Gavgani:** Data curation; Formal analysis; Investigation; Methodology; Software; Writing‐review & editing. **Eilnaz Basardeh** **:** Data curation; Formal analysis; Investigation; Methodology; Writing‐review & editing. **Mostafa Ghanei:** Conceptualization; Resources. **Masoumeh Azizi** **:** Formal analysis; Software. **Zabihollah Khaksar:** Investigation. **Zahra Sharifzadeh** **:** Data curation; Formal analysis. **Farzad Badmasti** **:** Investigation. **Mahdiyeh Soezi** **:** Investigation; Methodology. **Abolfazl Fateh:** Software. **Parisa Azimi** **:** Methodology. **Seyed Davar Siadat:** Conceptualization. **Fahimeh Shooraj:** Methodology. **Saeid Bouzari:** Conceptualization. **Mir Davoud Omrani** **:** Conceptualization; Resources. **Fatemeh Rahimi‐Jamnani:** Conceptualization; Data curation; Formal analysis; Funding acquisition; Investigation; Methodology; Project administration; Resources; Software; Supervision; Validation; Writing‐original draft; Writing‐review & editing.
