# Supplementary material for: Bactericidal fully human single‐chain fragment variable antibodies protect mice against methicillin‐resistant Staphylococcus aureus bacteraemia
Source: Clin Transl Immunology. 2021 Jun 29;10(7):e1302. doi: 10.1002/cti2.1302 (PMC8240403; doi:10.1002/cti2.1302)
Supplement: Supplementary file 1 [file CTI2-10-e1302-s001.pdf]

Supporting information

Supplementary Figures

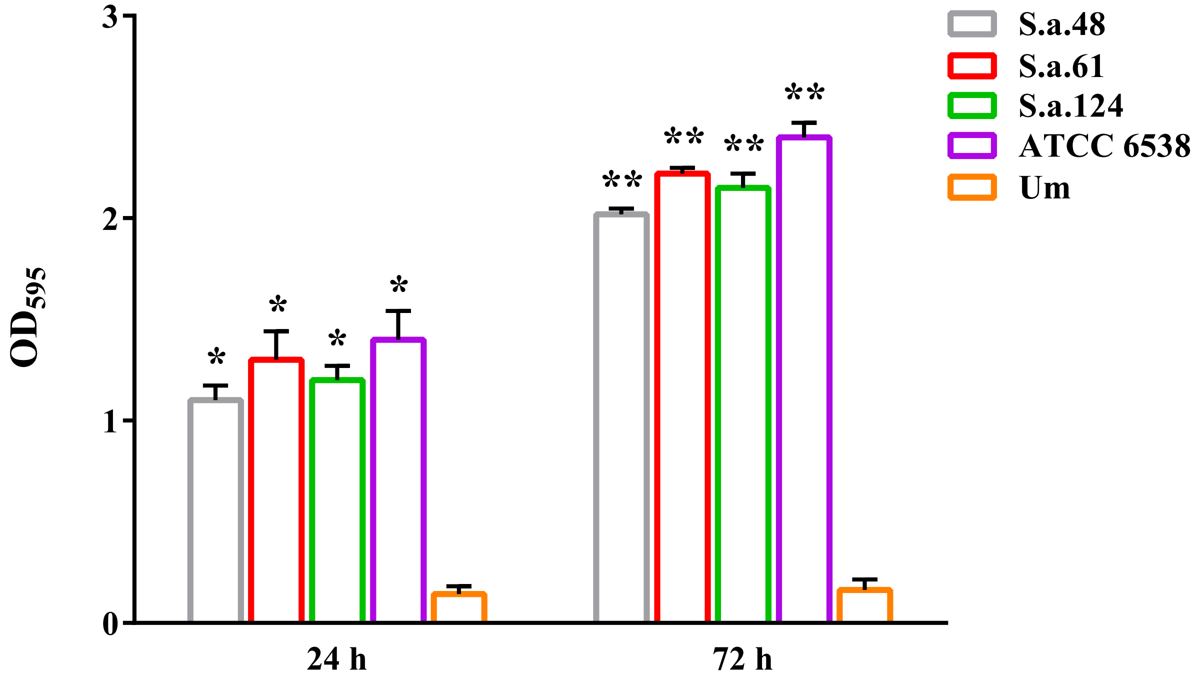

Supplementary figure 1. All four *S. aureus* strains (*S. aureus* S.a.48, S.a.61, S.a.124, and ATCC 6538) developed strong biofilms after 24 and 72 hours. The uninoculated medium (Um) is a negative control. Data are representative of three independent experiments, and error bars correspond to the mean ± SD. * *P*-value < 0.01, ** *P*-value < 0.001.

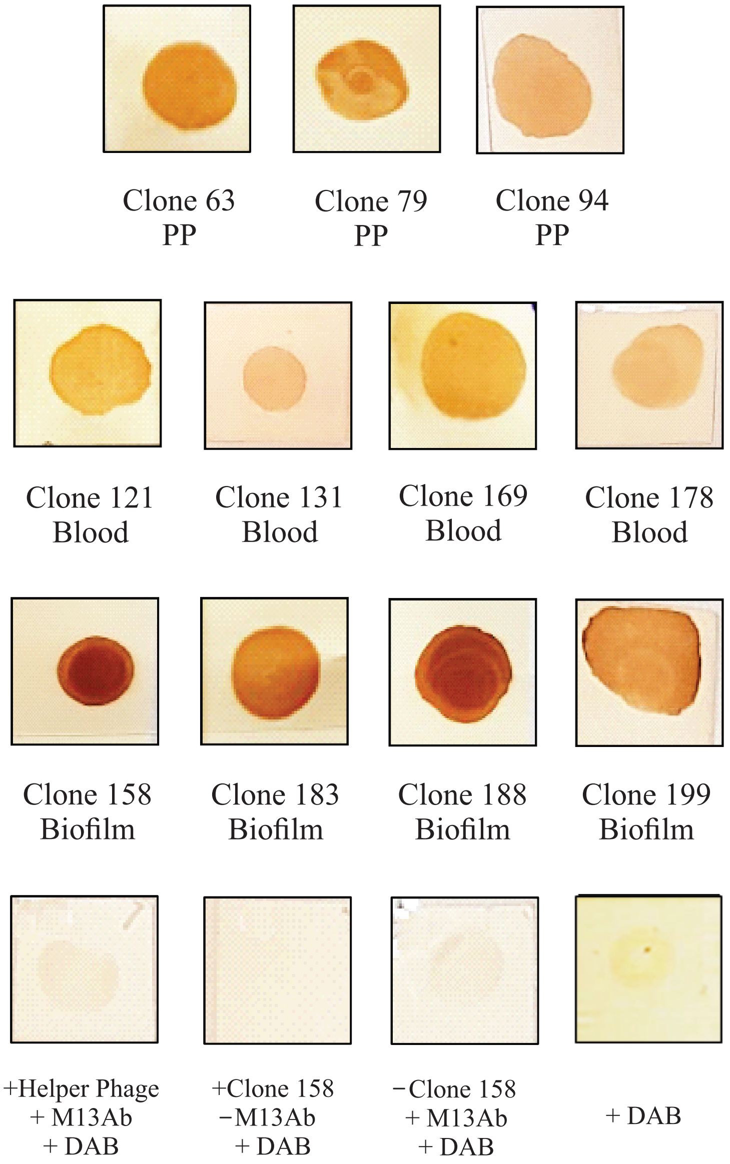

Supplementary figure 2. Eleven phage clones could bind *S. aureus*. The binding ability of 11 phage clones to *S. aureus* S.a.124 was appraised by the dot blot assay. The phages displaying scFvs (~ 10^{12} CFU mL^{-1}) could bind to *S. aureus* (~ 10^{8} CFU mL^{-1}) after probing with horseradish peroxidase (HRP)-conjugated anti-M13 major coat protein antibody (M13Ab). A group of controls was included, that is, *S. aureus* bacteria incubated with helper phages (instead of phage-scFvs; ~ 10^{12} CFU mL^{-1}) and then M13Ab; phages purified from the phage clone 158, but not incubated with M13Ab; M13Ab, and *S. aureus* bacteria incubated directly with DAB.

Biofilm: biofilm biopanning, Blood: blood biopanning, DAB: diaminobenzidine, PBMCs: peripheral blood mononuclear cells biopanning, PP: PBMC-plasma biopanning.

MEH63

FR1 CDR1 FR2 CDR2
QVQLVQSGAEVKKPGASVKVSCKASGYRLTSYEMHWVRQAPGQGLEWMGII-P-W-ST

FR3 CDR3 FR4
SYAQKFQGRVTMTRDTSTSTVYMELSSLRSEDTAVYYC-R-N-HG-L-A-DIWGQGTLVTVSSLE

GGGGSGGGGSGGGAS

FR1 CDR1 FR2 CDR2
DIQMTQSPSSLSASVGDRVTITCRASQSNYIGLNWYHQKPGKAPKLLIS-A-G

FR3 CDR3 FR4
SLQSGVPSRFSGSGSGTDFTLTISSLQPEDFATYYCQ-SY-R-A-FGGGTKVDIKRAAA

MEH158

FR1 CDR1 FR2 CDR2
QVQLVQSGAEVKKPGASVKVSCKASGYTFTSYEMHWVRQAPGQGLEWMGII--S-A-T

FR3 CDR3 FR4
SYAQKFQGRVTMTRDTSTSTVYMELSSLRSEDTAVYYCR-G-GH-S-WA--IWGQGTLVTVSSLE

GGGGSGGGGSGGGAS

FR1 CDR1 FR2 CDR2
DIQMTQSPSSLSASVGDRVTITCRASGQSINYLNWYHQKPGKAPKLLISG-SR

FR3 CDR3 FR4
SLQSGVPSRFSGSGSGTDFTLTISSLQPEDFATYYCQR-YS-R-TFGGGTKVDIKRAAA

MEH183

FR1 CDR1 FR2 CDR2
QVQLVQSGAEVKKPGASVKVSCKASPYTFTSYEMHWVRQAPGQGLEWMGI-N-S-GNT

FR3 CDR3 FR4
SYAQKFQGRVTMTRDTSTSTVYMELSSLRSEDTAVYYCA-Y-T-G-E--FDIWGQGTLVTVSSLE

GGGGSGGGGSGGGAS

FR1 CDR1 FR2 CDR2
DIQMTQSPSSLSASVGDRVTITCRASQARPNYLNWYHQKPGKAPKLLISG-S-

FR3 CDR3 FR4
SLQSGVPSRFSGSGSGTDFTLTISSLQPEDFATYYCE-W-T-R-TFGGGTKVDIKRAAA

Supplementary figure 3. The amino acid sequence of MEH63, MEH158, and MEH183.

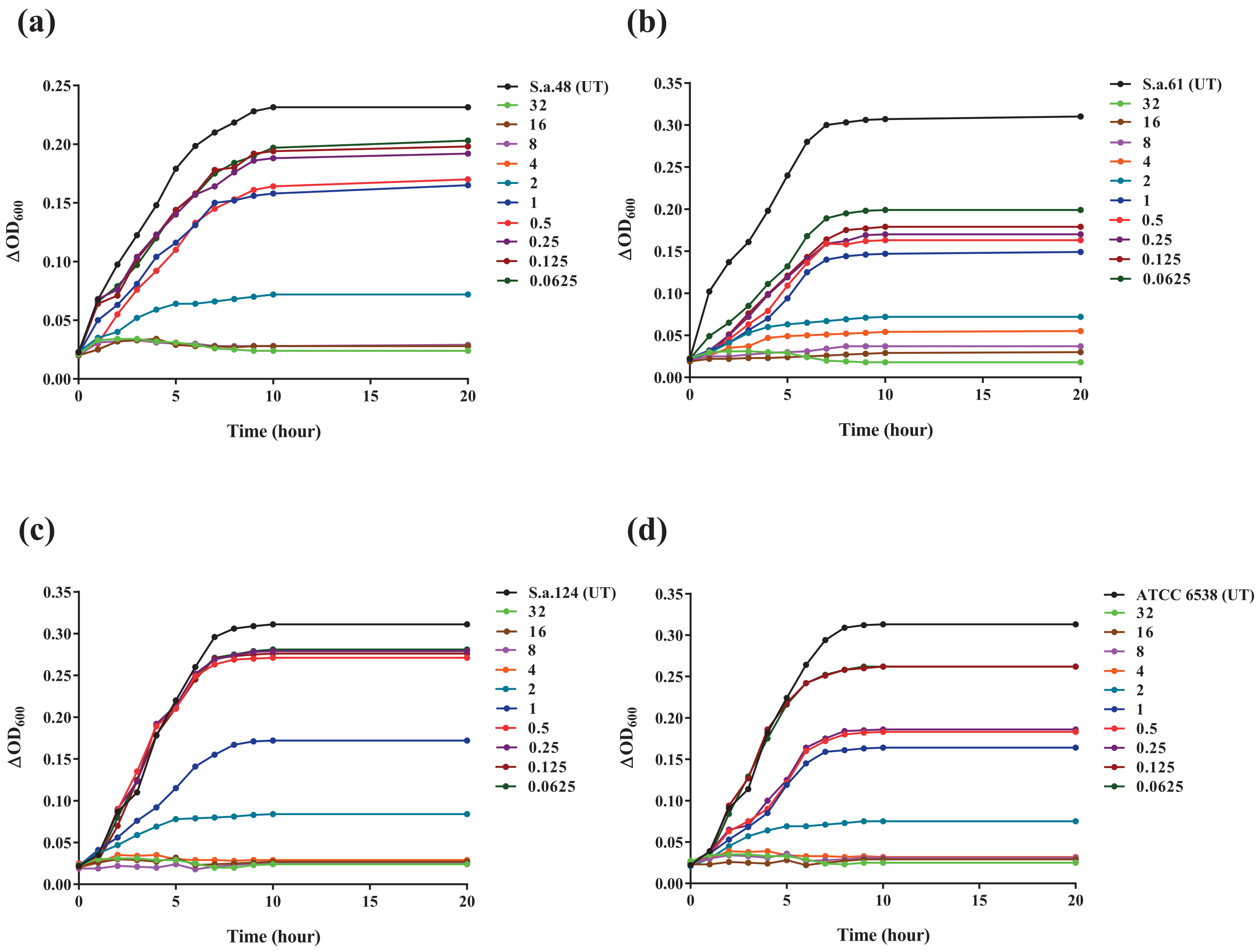

Supplementary figure 4. Vancomycin exhibited a significant inhibitory effect on the growth of *S. aureus* S.a.48, S.a.61, S.a.124, and ATCC 6538. In the microtiter plate assay, *S. aureus* **(a)** S.a.48, **(b)** S.a.61, **(c)** S.a.124, and **(d)** ATCC 6538 were incubated with vancomycin at concentrations ranging from 0.0625 to 32 μg mL^{-1}. The OD_{600} was determined every hour for 10 hours and once after 20 hours. Samples were run in triplicate and are representative of three independent experiments.

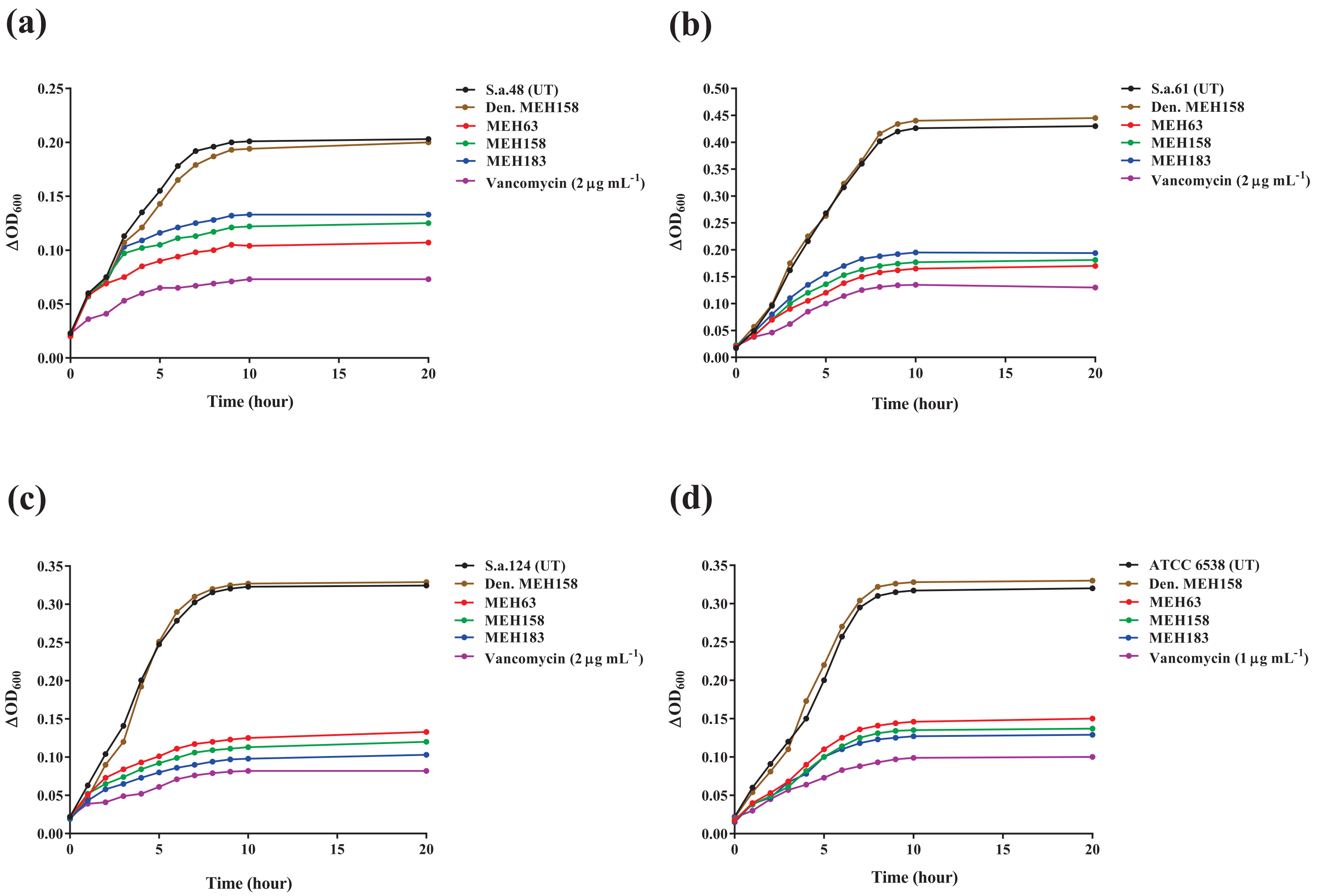

Supplementary figure 5. MEH63, MEH158, and MEH183 showed growth inhibitory activities against *S. aureus*. The antibacterial activity of MEH63, MEH158, and MEH183 against *S. aureus* was assessed by the microtiter plate assay. The growth curves of *S. aureus* **(a)** S.a.48, **(b)** S.a.61, **(c)** S.a.124, and **(d)** ATCC 6538, treated with the scFv (MEH63, MEH158, or MEH183; 200 μg mL^{-1}), vancomycin, or PBS. Samples were run in triplicate and are representative of three independent experiments.

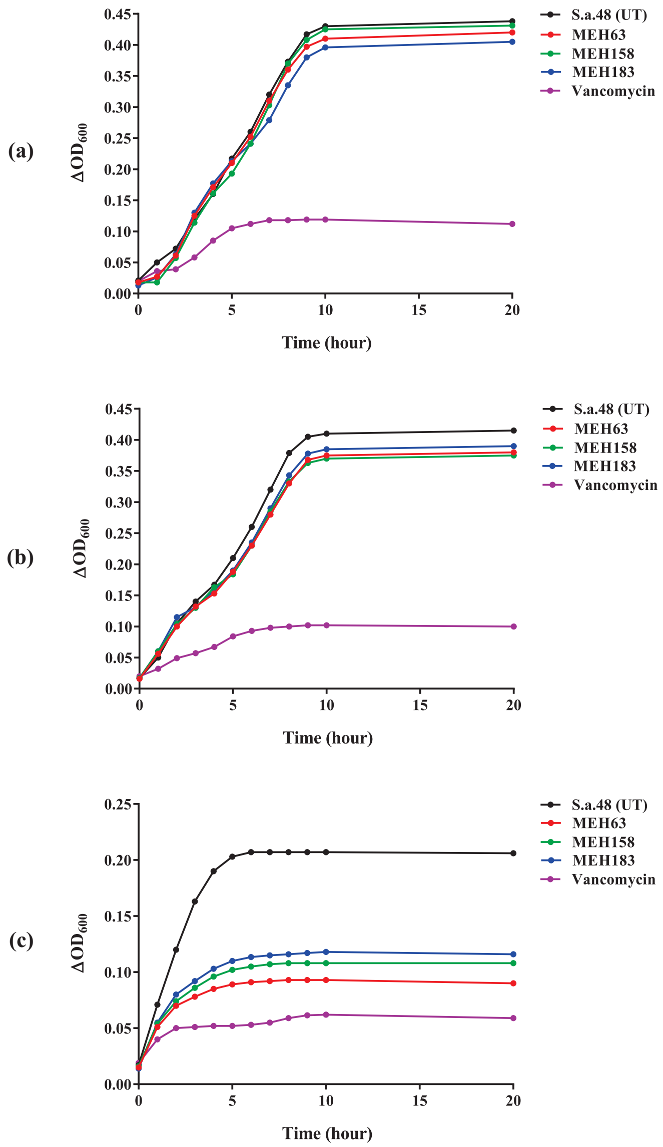

Supplementary figure 6. MEH63, MEH158, and MEH183 lost their growth inhibitory effects on *S. aureus* S.a.48 in the presence of high concentrations of Mg^{2+}. The antibacterial activity of MEH63, MEH158, and MEH183 against *S. aureus* S.a.48 in the presence of high concentrations of Mg^{2+} was assessed by the microtiter plate assay. The inhibitory activity of the scFvs against the growth of bacteria was diminished in the presence of 5 and 20 mM $MgSO_4$. The growth curves of bacteria treated with the scFv (MEH63, MEH158, or MEH183; 200 µg mL^{-1}), vancomycin (2 µg mL^{-1}), or PBS is illustrated in the presence of **(a)** 5 mM and **(b)** 20 mM $MgSO_4$. **(c)** The growth curves of bacteria incubated with the scFv (200 µg mL^{-1}), vancomycin (2 µg mL^{-1}), or PBS in the absence of $MgSO_4$. Samples were run in triplicate and are representative of three independent experiments.

(a)

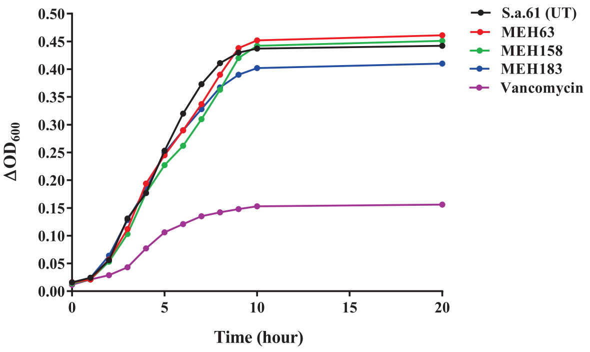

(b)

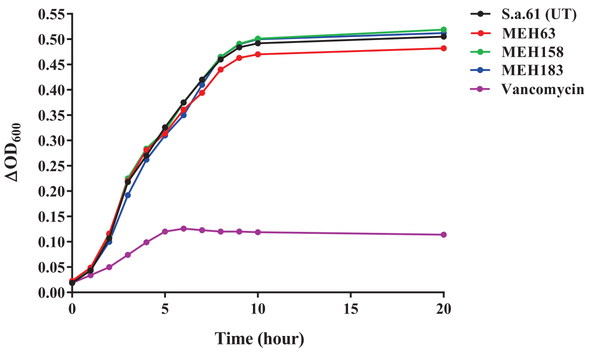

(c)

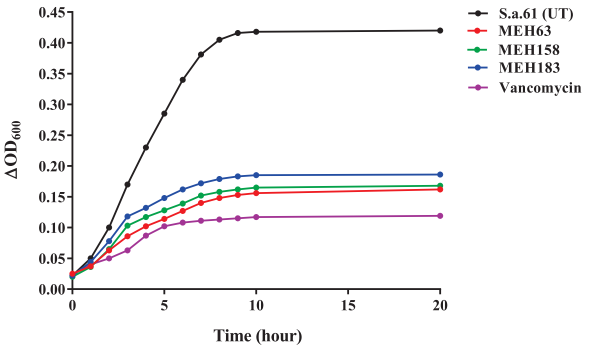

Supplementary figure 7. MEH63, MEH158, and MEH183 lost their growth inhibitory effects on *S. aureus* S.a.61 in the presence of high concentrations of Mg^{2+}. The antibacterial activity of MEH63, MEH158, and MEH183 against *S. aureus* S.a.61 in the presence of high concentrations of Mg^{2+} was assessed by the microtiter plate assay. The inhibitory activity of the scFvs against the growth of bacteria was diminished in the presence of 5 and 20 mM $MgSO_4$. The growth curves of bacteria treated with the scFv (MEH63, MEH158, or MEH183; 200 µg mL^{-1}), vancomycin (2 µg mL^{-1}), or PBS is illustrated in the presence of **(a)** 5 mM and **(b)** 20 mM $MgSO_4$. **(c)** The growth curves of bacteria incubated with the scFv (200 µg mL^{-1}), vancomycin (2 µg mL^{-1}), or PBS in the absence of $MgSO_4$. Samples were run in triplicate and are representative of three independent experiments.

(a)

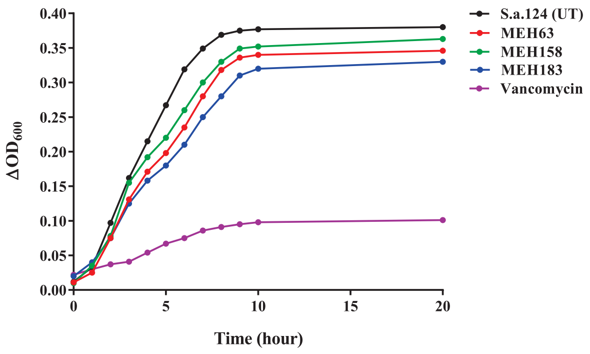

(b)

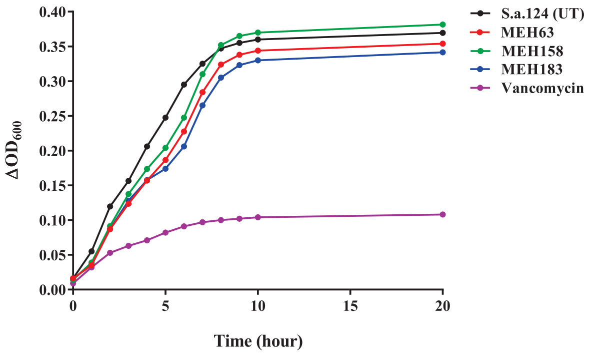

(c)

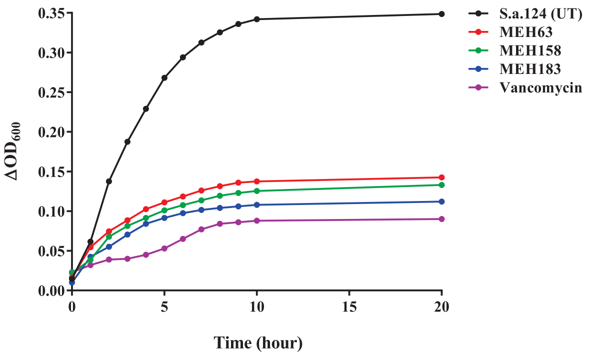

Supplementary figure 8. MEH63, MEH158, and MEH183 lost their growth inhibitory effects on *S. aureus* S.a.124 in the presence of high concentrations of Mg^{2+}. The antibacterial activity of MEH63, MEH158, and MEH183 against *S. aureus* S.a.124 in the presence of high concentrations of Mg^{2+} was assessed by the microtiter plate assay. The inhibitory activity of the scFvs against the growth of bacteria was diminished in the presence of 5 and 20 mM $MgSO_4$. The growth curves of bacteria treated with the scFv (MEH63, MEH158, or MEH183; 200 µg mL^{-1}), vancomycin (2 µg mL^{-1}), or PBS in the presence of **(a)** 5 mM and **(b)** 20 mM $MgSO_4$. **(c)** The growth curves of bacteria incubated with the scFv (200 µg mL^{-1}), vancomycin (2 µg mL^{-1}), or PBS in the absence of $MgSO_4$. Samples were run in triplicate and are representative of three independent experiments.

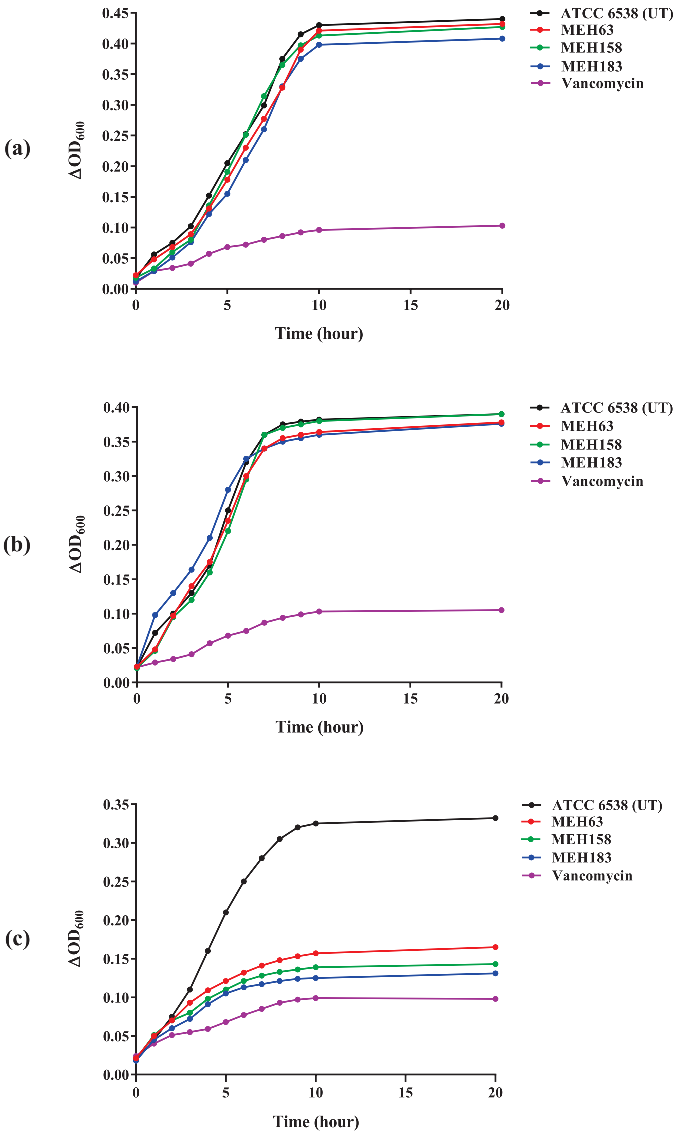

Supplementary figure 9. MEH63, MEH158, and MEH183 lost their growth inhibitory effects on *S. aureus* ATCC 6538 in the presence of high concentrations of Mg^{2+}. The antibacterial activity of MEH63, MEH158, and MEH183 against *S. aureus* ATCC 6538 in the presence of high concentrations of Mg^{2+} was assessed by the microtiter plate assay. The inhibitory activity of the scFvs against the growth of bacteria was diminished in the presence of 5 and 20 mM $MgSO_4$. The growth curves of bacteria treated with the scFv (MEH63, MEH158, or MEH183; 200 μg mL^{-1}), vancomycin (1 μg mL^{-1}), or PBS is illustrated in the presence of **(a)** 5 mM and **(b)** 20 mM $MgSO_4$. **(c)** The growth curves of bacteria incubated with the scFv (200 μg mL^{-1}), vancomycin (1 μg mL^{-1}), or PBS in the absence of $MgSO_4$. Samples were run in triplicate and are representative of three independent experiments.

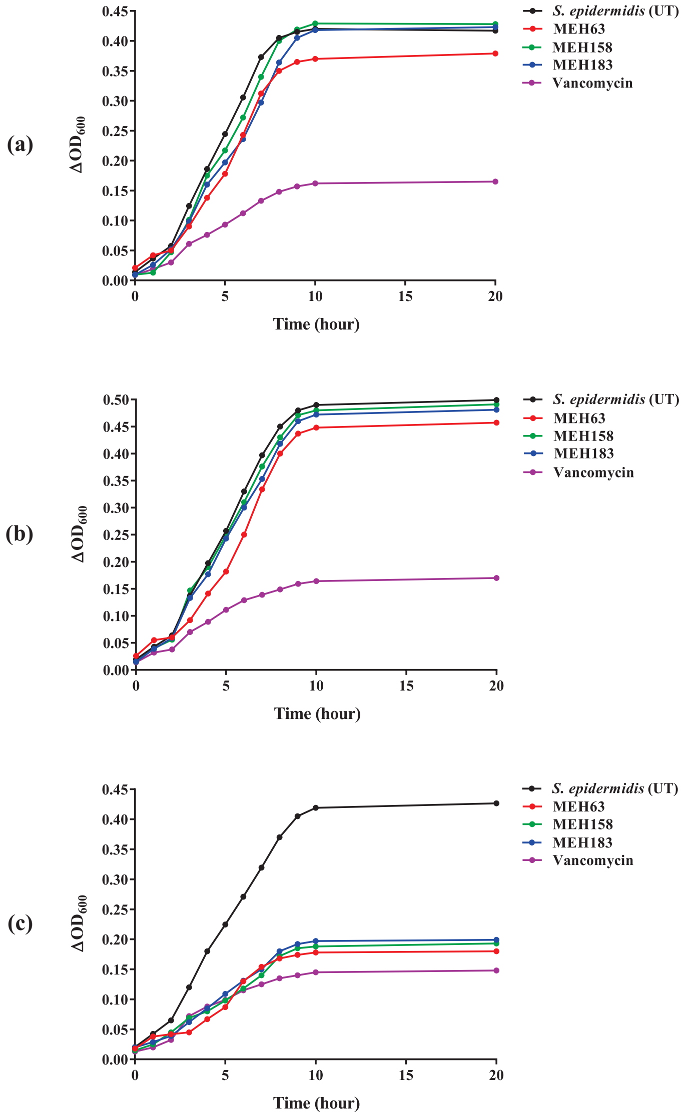

Supplementary figure 10. MEH63, MEH158, and MEH183 lost their growth inhibitory effects on *S. epidermidis* in the presence of high concentrations of Mg^{2+}. The antimicrobial activity of MEH63, MEH158, and MEH183 against *S. epidermidis* ATCC 12228 was assessed in the presence of high concentrations of Mg^{2+} by the microtiter plate assay. The inhibitory activity of the scFvs against the growth of bacteria was diminished in the presence of 5 and 20 mM $MgSO_4$. The growth curves of bacteria treated with the scFv (MEH63, MEH158, or MEH183; 200 µg mL^{-1}), vancomycin (1 µg mL^{-1}) or PBS in the presence of **(a)** 5 mM and **(b)** 20 mM $MgSO_4$. **(c)** The growth curves of bacteria incubated with the scFv (200 µg mL^{-1}), vancomycin (1 µg mL^{-1}), or PBS in the absence of $MgSO_4$. Samples were run in triplicate and are representative of three independent experiments.

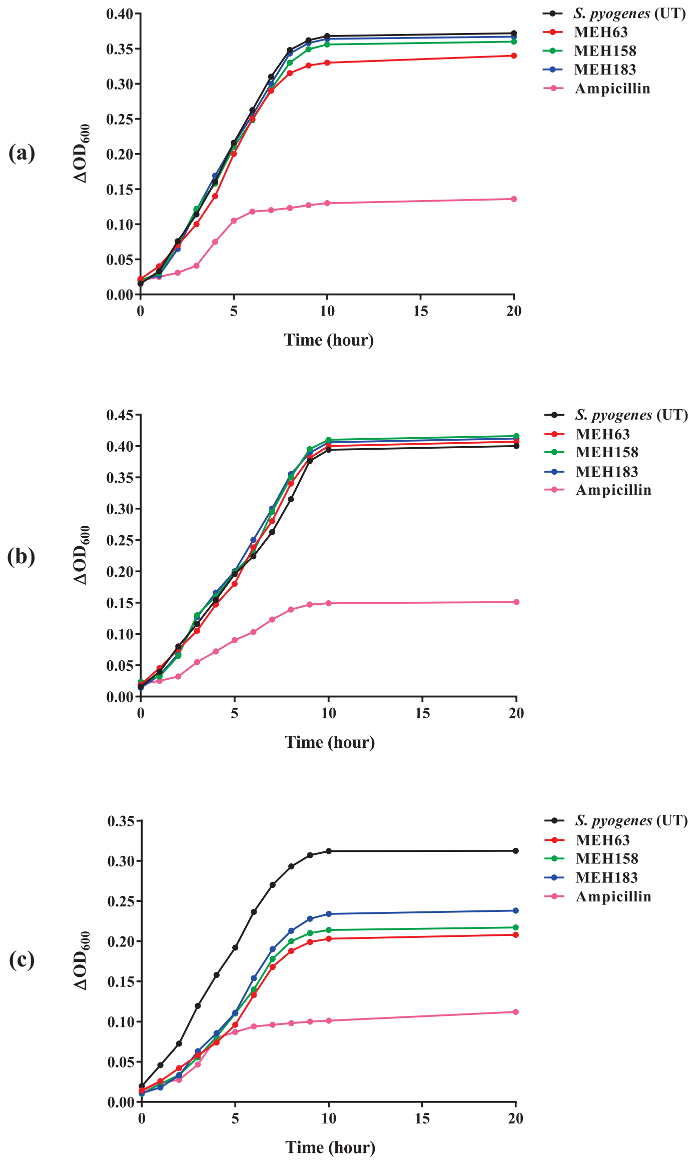

Supplementary figure 11. MEH63, MEH158, and MEH183 lost their growth inhibitory effects on *Streptococcus pyogenes* in the presence of high concentrations of Mg^{2+}. The antimicrobial activity of MEH63, MEH158, and MEH183 against *S. pyogenes* ATCC 10403 was assessed in the presence of high concentrations of Mg^{2+} by the microtiter plate assay. The inhibitory activity of the scFvs against the growth of bacteria was diminished in the presence of 5 and 20 mM $MgSO_4$. The growth curves of bacteria treated with the scFv (MEH63, MEH158, or MEH183; 200 µg mL^{-1}), ampicillin (0.5 µg mL^{-1}), or PBS in the presence of **(a)** 5 mM and **(b)** 20 mM $MgSO_4$. **(c)** The growth curves of bacteria incubated with the scFv (200 µg mL^{-1}), ampicillin (0.5 µg mL^{-1}), or PBS in the absence of $MgSO_4$. Samples were run in triplicate and are representative of three independent experiments.

(a)

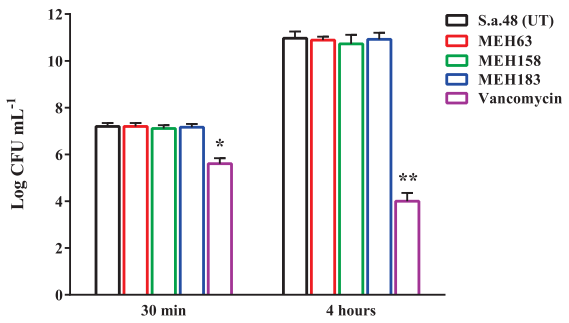

(b)

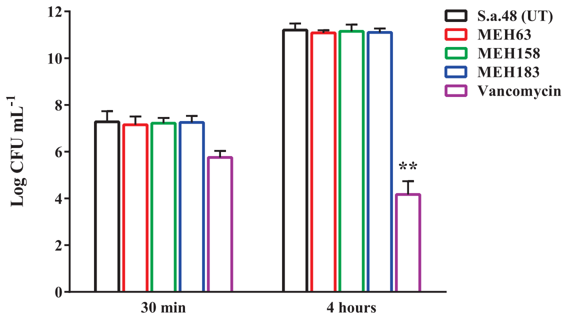

(c)

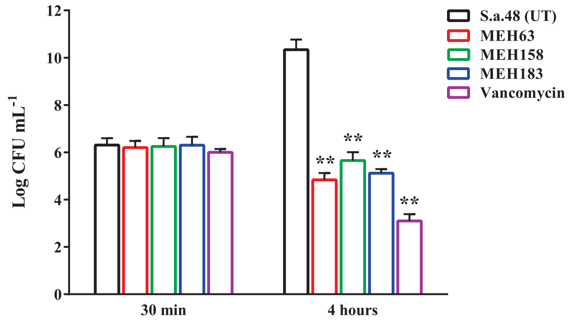

Supplementary figure 12. The antibacterial activity of MEH63, MEH158, and MEH183 against *S. aureus* S.a.48 was diminished in the presence of high concentrations of Mg^{2+}. The bactericidal activity of MEH63, MEH158, and MEH183 against *S. aureus* S.a.48 was assessed in the presence of high concentrations of Mg^{2+} by the agar plate assay. MEH63, MEH158, and MEH183 showed no inhibitory activities against *S. aureus* S.a.48 in the presence of **(a)** 5 mM and **(b)** 20 mM $MgSO_4$ at 30 min and four hours of incubation. **(c)** MEH63, MEH158, and MEH183 could significantly reduce the CFU count in the absence of Mg^{2+} at four hours of incubation. Vancomycin (2 µg mL^{-1}) exhibited marked antibacterial activity in the absence and presence of Mg^{2+} at four hours of incubation. Data are representative of three independent experiments, and error bars correspond to the mean ± SEM. * *P*-value < 0.05, ** *P*-value < 0.01.

(a)

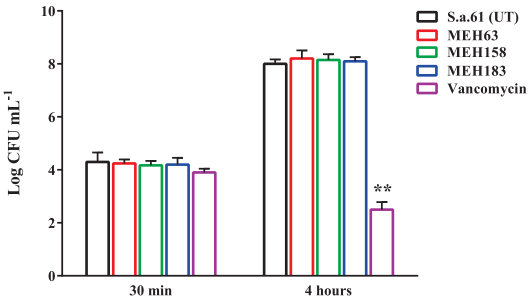

(b)

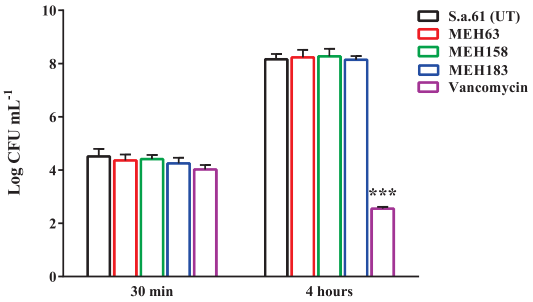

(c)

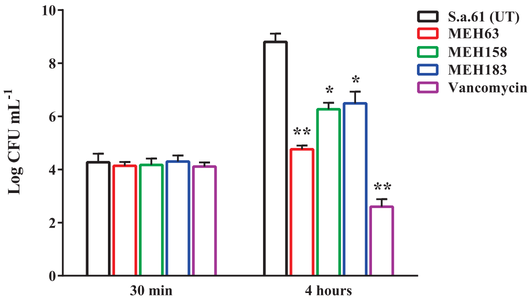

Supplementary figure 13. The antibacterial activity of MEH63, MEH158, and MEH183 against *S. aureus* S.a.61 was diminished in the presence of high concentrations of Mg^{2+}. The bactericidal activity of MEH63, MEH158, and MEH183 against *S. aureus* S.a.61 was assessed in the presence of high concentrations of Mg^{2+} by the agar plate assay. MEH63, MEH158, and MEH183 showed no inhibitory activities against *S. aureus* S.a.61 in the presence of **(a)** 5 mM and **(b)** 20 mM $MgSO_4$ at 30 min and four hours of incubation. **(c)** MEH63, MEH158, and MEH183 could significantly reduce the CFU count in the absence of Mg^{2+} at four hours of incubation. Vancomycin (2 μg mL^{-1}) exhibited marked antibacterial activity in the absence and presence of Mg^{2+} at four hours of incubation. Data are representative of three independent experiments, and error bars correspond to the mean ± SEM. * *P*-value < 0.05, ** *P*-value < 0.01, *** *P*-value < 0.001.

(a)

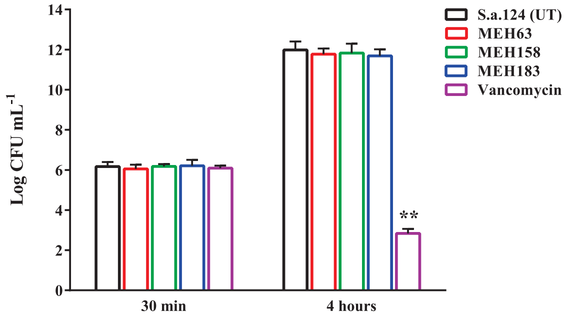

(b)

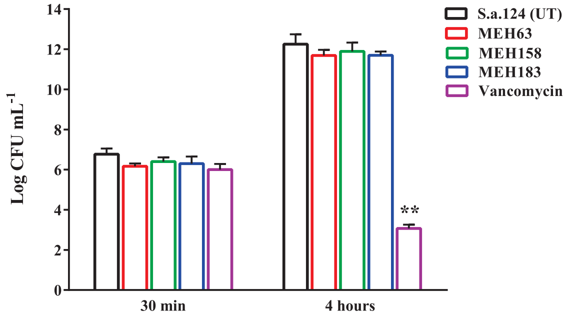

(c)

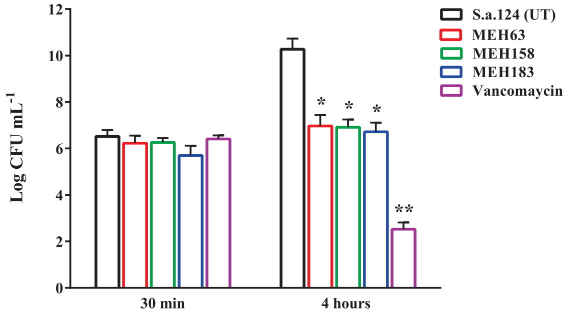

Supplementary figure 14. The antibacterial activity of MEH63, MEH158, and MEH183 against *S. aureus* S.a.124 was diminished in the presence of high concentrations of Mg^{2+}. The bactericidal activity of MEH63, MEH158, and MEH183 against *S. aureus* S.a.124 was assessed in the presence of high concentrations of Mg^{2+} by the agar plate assay. MEH63, MEH158, and MEH183 showed no inhibitory activities against *S. aureus* S.a.124 in the presence of **(a)** 5 mM and **(b)** 20 mM $MgSO_4$ at 30 min and four hours of incubation. **(c)** MEH63, MEH158, and MEH183 could significantly reduce the CFU count in the absence of Mg^{2+} at four hours of incubation. Vancomycin (2 µg mL^{-1}) exhibited marked antibacterial activity in the absence and presence of Mg^{2+} at four hours of incubation. Data are representative of three independent experiments and error bars correspond to the mean ± SEM. * *P*-value < 0.05, ** *P*-value < 0.01

(a)

(b)

(c)

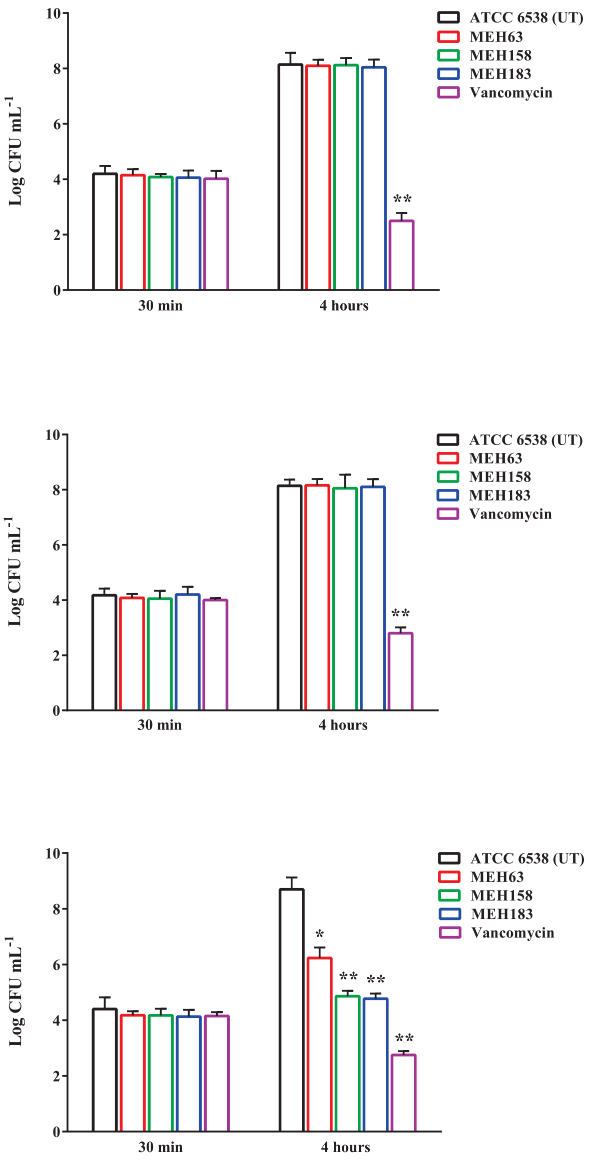

Supplementary figure 15. The antibacterial activity of MEH63, MEH158, and MEH183 against *S. aureus* ATCC 6538 was diminished in the presence of high concentrations of Mg^{2+}. The bactericidal activity of MEH63, MEH158, and MEH183 against *S. aureus* ATCC 6538 was assessed in the presence of high concentrations of Mg^{2+} by the agar plate assay. MEH63, MEH158, and MEH183 showed no inhibitory activities against *S. aureus* ATCC 6538 in the presence of **(a)** 5 mM and **(b)** 20 mM $MgSO_4$ at 30 min and four hours of incubation. **(c)** MEH63, MEH158, and MEH183 could significantly reduce the CFU count in the absence of Mg^{2+} at four hours of incubation. Vancomycin (1 µg mL^{-1}) exhibited marked antibacterial activity in the absence and presence of Mg^{2+} at four hours of incubation. Data are representative of three independent experiments, and error bars correspond to the mean ± SEM. * P-value < 0.05, ** P-value < 0.01

(a)

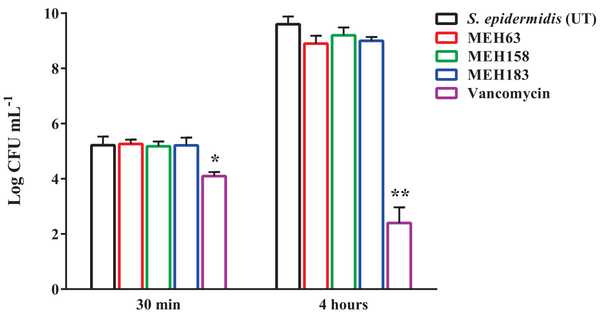

(b)

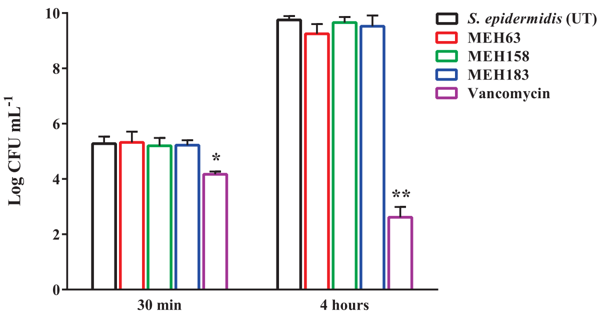

(c)

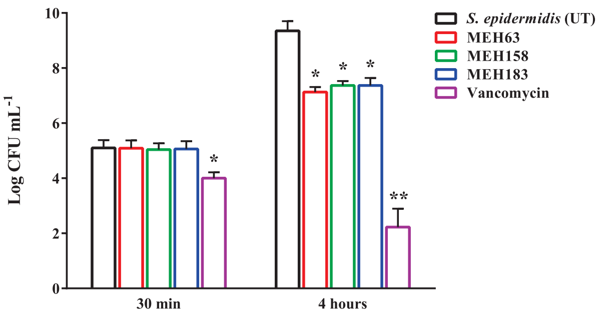

Supplementary figure 16. The antibacterial activity of MEH63, MEH158, and MEH183 against *S. epidermidis* was diminished in the presence of high concentrations of Mg^{2+}. The inhibitory effect of MEH63, MEH158, and MEH183 on the growth of *S. epidermidis* ATCC 12228 was assessed in the presence of high concentrations of Mg^{2+} by the agar plate assay. MEH63, MEH158, and MEH183 showed no inhibitory activities against *S. epidermidis* ATCC 12228 in the presence of **(a)** 5 mM and **(b)** 20 mM $MgSO_4$ at 30 min and four hours of incubation. **(c)** MEH63, MEH158, and MEH183 could significantly reduce the CFU count in the absence of Mg^{2+} at four hours of incubation. Vancomycin (1 μg mL^{-1}) exhibited antibacterial activity in the absence and presence of Mg^{2+} at four hours of incubation. Data are representative of three independent experiments and error bars correspond to the mean ± SEM. * *P*-value < 0.05, ** *P*-value < 0.01

(a)

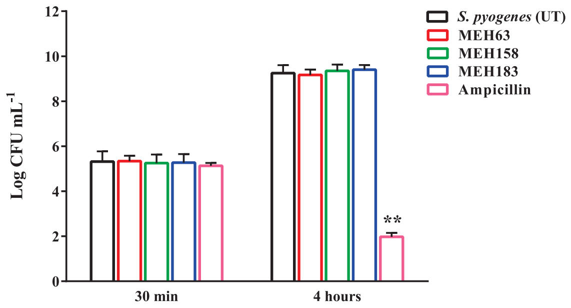

(b)

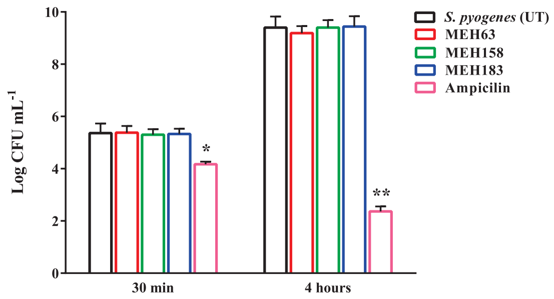

(c)

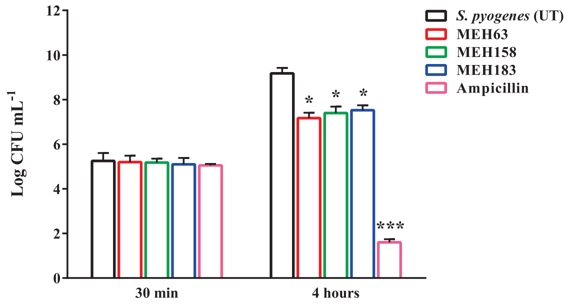

Supplementary figure 17. The antibacterial activity of MEH63, MEH158, and MEH183 against *S. pyogenes* was diminished in the presence of high concentrations of Mg^{2+}. The inhibitory effect of MEH63, MEH158, and MEH183 *S. pyogenes* ATCC 10403 was assessed in the presence of high concentrations of Mg^{2+} by the agar plate assay. MEH63, MEH158, and MEH183 showed no inhibitory activities against *S. pyogenes* ATCC 10403 in the presence of **(a)** 5 mM and **(b)** 20 mM $MgSO_4$ at 30 min and four hours of incubation. **(c)** MEH63, MEH158, and MEH183 could significantly reduce the CFU count in the absence of Mg^{2+} at four hours of incubation. Ampicillin (0.5 µg mL^{-1}) exhibited antibacterial activity in the absence and presence of Mg^{2+} at four hours of incubation. Data are representative of three independent experiments, and error bars correspond to the mean ± SEM. * P-value < 0.05, ** P-value < 0.01, *** P-value < 0.001.

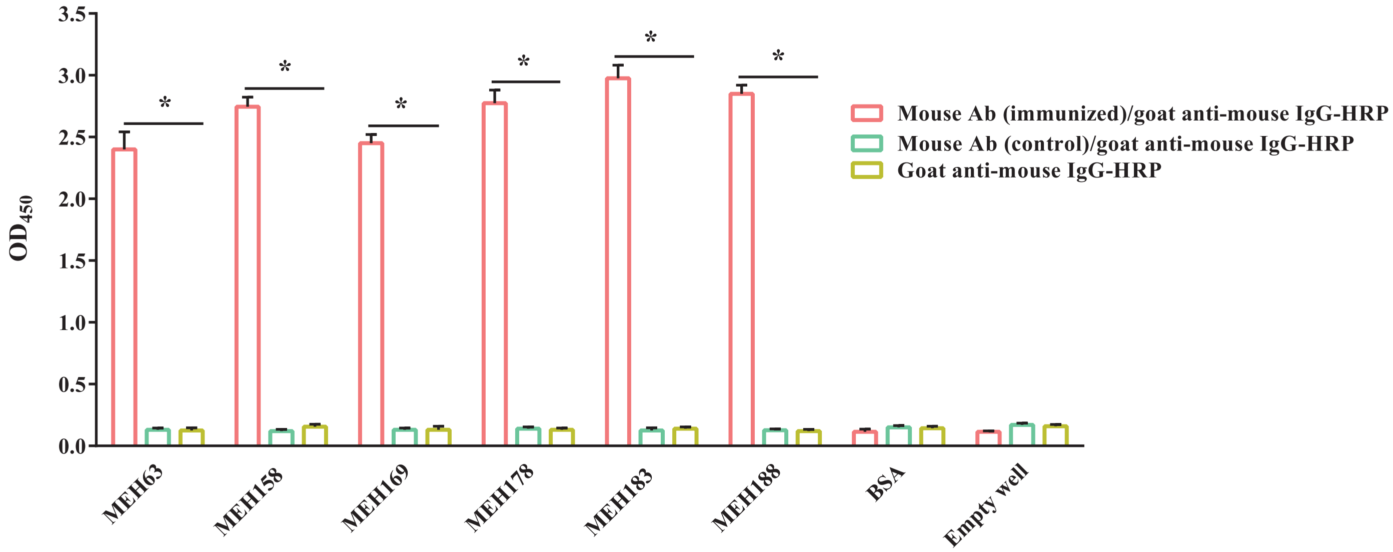

Supplementary figure 18. Mouse anti-human scFv antibodies showed strong binding to the selected scFvs. To produce mouse anti-human scFv antibodies, female BALB/c mice (six per group, six to eight weeks old), supplied by the Animal Laboratory of Pasteur Institute of Iran, were injected 5 µg per gram of purified scFvs (MEH63, MEH178, and MEH183) intraperitoneally. The mice receiving normal saline served as the control group. After 10 days, the blood of mice was collected, and immunoglobulin G (IgG) purification was performed using Protein G Mag Sepharose Xtra beads (GE Healthcare). The purity and binding ability of IgGs were evaluated by sodium dodecyl sulfate-polyacrylamide gel electrophoresis (SDS-PAGE) and enzyme-linked immunosorbent assay (ELISA), respectively. In ELISA, the wells of a 96-well plate were coated with the scFv antibody (MEH63, MEH158, MEH169, MEH178, MEH183, and MEH188) (1 µg mL^{-1}) or bovine serum albumin (BSA) (5 mg mL^{-1}). After blocking, the wells were incubated with mouse anti-human scFv antibody (mouse Ab; immunized) or IgG of the control group (mouse Ab; control) (1:200 dilution) for one hour at RT. After several times washing with PBS plus 0.05% Tween-20 (PBST), goat anti-mouse IgG-horseradish peroxidase (HRP) antibody (1:2000 dilution) was added to the wells and incubated for one hour at RT, followed by washing and addition of 3, 3′, 5, 5′-tetramethylbenzidine (TMB). Next, the developing reactions were terminated by sulfuric acid (H_2SO_4) solution, and absorbance was read at OD_{450} by a microtiter plate reader. Results are representative of three independent experiments, and error bars correspond to the mean ± SEM. Statistical comparisons were performed by two-way analysis of variance (ANOVA). * $P < 0.0001$.
